# Exploiting epigenetic targets to overcome taxane resistance in prostate cancer

**DOI:** 10.1038/s41419-024-06422-1

**Published:** 2024-02-12

**Authors:** Buse Cevatemre, Ipek Bulut, Beyza Dedeoglu, Arda Isiklar, Hamzah Syed, Ozlem Yedier Bayram, Tugba Bagci-Onder, Ceyda Acilan

**Affiliations:** 1 Koc University Research Center for Translational Medicine, Istanbul, Turkey; 2 Koc University Graduate School of Health Sciences, Istanbul, Turkey; 3 Koc University Graduate School of Science and Engineering, Istanbul, Turkey; 4 Koc University School of Medicine, Sariyer, Turkey

**Keywords:** Urological cancer, Cancer therapeutic resistance, Chemotherapy, Apoptosis, Phenotypic screening

## Abstract

The development of taxane resistance remains a major challenge for castration resistant prostate cancer (CR-PCa), despite the effectiveness of taxanes in prolonging patient survival. To uncover novel targets, we performed an epigenetic drug screen on taxane (docetaxel and cabazitaxel) resistant CR-PCa cells. We identified BRPF reader proteins, along with several epigenetic groups (CBP/p300, Menin-MLL, PRMT5 and SIRT1) that act as targets effectively reversing the resistance mediated by ABCB1. Targeting BRPFs specifically resulted in the resensitization of resistant cells, while no such effect was observed on the sensitive compartment. These cells were successfully arrested at the G_2_/M phase of cell cycle and underwent apoptosis upon BRPF inhibition, confirming the restoration of taxane susceptibility. Pharmacological inhibition of BRPFs reduced ABCB1 activity, indicating that BRPFs may be involved in an efflux-related mechanism. Indeed, ChIP-qPCR analysis confirmed binding of BRPF1 to the ABCB1 promoter suggesting direct regulation of the ABCB1 gene at the transcriptional level. RNA-seq analysis revealed that BRPF1 knockdown affects the genes enriched in mTORC1 and UPR signaling pathways, revealing potential mechanisms underlying its functional impact, which is further supported by the enhancement of taxane response through the combined inhibition of ABCB1 and mTOR pathways, providing evidence for the involvement of multiple BRPF1-regulated pathways. Beyond clinical attributes (Gleason score, tumor stage, therapy outcome, recurrence), metastatic PCa databases further supported the significance of BRPF1 in taxane resistance, as evidenced by its upregulation in taxane-exposed PCa patients.

## Introduction

Prostate cancer (PCa) is the second most common malignancy among men worldwide, comprising 13.5% of all male cancer diagnoses [1]. In early stages of the disease, prostatectomy and radiotherapy are considered as the main therapeutic options. However, 20–30% of patients relapse after 5–10 years, and Androgen Deprivation Therapy (ADT) is currently the primary approach for advanced disease. Although the majority of patients initially respond to ADT, PCa relapse is frequently observed in a few years leading to progression to the castration-resistant PCa (CR-PCa) stage [2, 3]. The major treatment option for metastatic CR-PCa includes the use of taxanes (mainly docetaxel, followed by cabazitaxel). Nevertheless, despite the prolonged survival resulting from docetaxel (Dtx), most patients become refractory due to the development of resistance and succumb to PCa. Thus, it is crucial to find alternative options to overcome resistance and resensitize patients to taxanes.

One of the major factors that contribute to drug resistance is alterations in the epigenome of cancer cells. Not surprisingly, epigenetic modifiers have been targeted by numerous researchers, and several clinical trials are currently recruiting patients to study the efficacy of epigenetic modifiers in PCa, including but not limited to inhibitors of bromodomain and extra-terminal domain (BET), histone methyltransferases (HMT), DNA methyltransferases (DNMT), histone deacetylases (HDACs) or CBP/p300 histone acetyltransferases (HATs) [4–6]. More importantly, some of these studies test epi-drugs in CR-PCa and progressive patients either as monotherapy or in combination with other drugs, including Dtx [7, 8]. These studies show that epigenetic drugs have the potential to overcome chemotherapy resistance in tumors.

Taxane resistance has been extensively investigated, revealing multiple mechanisms involved, such as upregulated taxane-metabolizing enzymes, prosurvival pathways, altered microtubule regulatory proteins, EMT induction, and dysregulated non-coding RNAs, among other identified contributors [9–12]. Despite the involvement of multiple pathways in the resistance phenotype, the major underlying factor is the drug efflux mediated by the ABCB1 transporter.

In this study, we established two Dtx- and cabazitaxel (Cbz)-resistant CR-PCa cells (Du145 and 22Rv1) and initially characterized the transcriptomic changes in these cells using RNA-seq analysis. Indeed, ABCB1 was one of the top hits, whose expression was greatly elevated. Interestingly, there was no increase in the copy number of ABCB1 gene in 3 out of 4 resistant cell lines, indicating that the transcriptional activity of ABCB1 was altered during the resistance process. Therefore, under the light of new developments in the PCa continuum, we assessed the role of epigenetic modifiers for their efficacy to revert taxane resistance in CR-PCa and conducted an epi-drug screen to uncover the resensitizing factors. We were able to identify five different groups of modulators (CBP/p300, Menin-MLL, SIRT, PRMT5 and BRPF) that were able to effectively revert resistance, all of which were verified via further analyses. Of these, BRPF inhibitors were effective when combined with taxanes for the resistant, but not for the sensitive parental cells. Silencing BRPF proteins showed a similar phenotype, supporting the importance of BRPFs for resistance. BRPF inhibition reduced ABCB1 activity and gene expression, suggesting the involvement of BRPFs in drug efflux. We observed the occupancy of the ABCB1 promoter by BRPF1 through ChIP-qPCR analysis, providing strong evidence for the direct involvement of BRPF1 in the regulation of the ABCB1 gene. By performing RNA-seq analysis following BRPF inhibition, we identified the modulation of genes associated with the mTORC1 and UPR signaling pathways. Silencing these pathways mimicked BRPF inhibition and reverted resistance, potentially explaining how BRPFs may overcome taxane resistance. Furthermore, the co-targeting of ABCB1 and mTOR pathways enhanced the response to taxanes, providing evidence for the involvement of multiple pathways under the regulation of BRPF1. Lastly, our analysis of BRPF1 expression in pan-cancer and PCa databases demonstrated its significant association with various clinical attributes, including the upregulation observed in taxane-exposed PCa patients, suggesting BRPF1 as a potential biomarker for the progression of PCa.

## Materials and methods

### Generation of taxane-resistant prostate cancer cell models

Du145 (ATCC no. HTB-81) and 22Rv1 (ATCC no. CRL-2505) cells were maintained in RPMI-1640 medium (Gibco, 11875093) supplemented with 10% fetal bovine serum (Biowest, S1810) and 1% penicillin/streptomycin (Biowest, L0022). Cells were grown at 37 °C in a humidified atmosphere with 5% CO_2_ and routinely tested for mycoplasma contamination. Resistant clones were selected by culturing cells with docetaxel (Sigma-Aldrich, 01885) and/or cabazitaxel (Sigma-Aldrich, SML2487) for 72 h. A dose-escalation strategy was implemented, beginning at 1 nM, and doubled to reach 125 nM. After waiting for approximately 1–2 weeks for the remaining survived cells to recover, they underwent a passage before proceeding with the new dose. Cell viability experiments were set up to assess the establishment of resistance at two dose increments. Parental cells were passaged alongside as an age-matched appropriate control.

### CRISPR-Cas9

gRNA sequences were designed using the Benching software (https://www.benchling.com) and are listed in Sup. Table 1. LentiCRISPR v2 (Addgene #52961) plasmid DNA (2 µg) was digested with BsmBI-v2 (NEB, R0739) at 55 °C for 3 h. The digested vector was dephosphorylated (NEB Antarctic Phosphatase, M0289S) for 30 min, purified using the NucleoSpin Gel and PCR Cleanup kit (Macherey-Nagel™, 740609) and then ligated with annealed gRNA oligos. To produce lentivirus, Hek293T cells were co-transfected with 2500 ng of the cloned plasmid, 2250 ng of the packaging psPAX2 (Addgene 12260) plasmid, and 250 ng of the envelope pVSVg (Addgene, 14888) plasmid using FuGENE®6 (Promega, E2691). The following day, the cell culture medium was refreshed and for the next three days, the virus-containing supernatant was collected and subsequently precipitated using PEG (Sigma, P2139). Viruses further precipitated by centrifugation, then diluted with PBS, aliquoted, and stored at −80 °C. Transduction was carried out using Polybrene (8 µg/ml). Cells were selected with puromycin (Sigma P7255, 10 µg/mL) for 4 days, and knockout was confirmed by western blotting.

### shRNA cloning

shRNA sequences were designed using the Broad Institute GPP (https://portals.broadinstitute.org/gpp/public/) and are listed in Sup. Table 1. The pLKO.1 (Addgene #8453) lentiviral vector was digested (2 µg) with AgeI (NEB, R3552) and EcoRI (NEB, R3101). Transfection, lentivirus production, and transduction were performed as described for CRISPR-Cas9.

### Cell proliferation and doubling time analysis

#### Sulforhodamine B viability assay

Du145 (4 × 10^3^) and 22Rv1 (7.5 × 10^4^) cells were seeded on 96-well plates the day prior to drug exposure. Cells were fixed with a final concentration of 10% (w/v) TCA (Sigma, T6399) at 4 °C for 1 h, stained with 0.4% (w/v) SRB (Santa Cruz, sc-253615) for 30 min and subsequently washed with 1% acetic acid. Sulforhodamine B (SRB) dye was extracted using a 10 mM Trizma base (Sigma, T1503). Measurements were carried out at 564 nm using a microplate reader (Synergy H1 Hybrid reader, BioTek). Epigenetics Screening Library (Cayman, 11076) was used to uncover the epigenetic regulators of taxane resistance. Du145-P/R (4 × 10^3^) and 22Rv1-P/R (1 × 10^4^) cells were co-treated with compounds (5 μM) and IC_25_ values of taxanes for 72 h. Cell viability normalization was performed as a percentage with the formula: % Cell Viability = (Treated Cell Absorbance−blank/Untreated Cell Absorbance−blank) × 100.

#### Colony formation assay

Du145 (7.5 × 10^2^) and 22Rv1 (1 × 10^3^) cells were seeded on 12-well plates the day prior to taxane exposure. At the end of the treatment, the medium was replaced by drug-free medium, and the cells were cultured for an additional 10 days. The colonies were fixed with methanol (Merck, 1.06009) at −20 °C for 10 min, stained with 0.5% (w/v) crystal violet (Merck, 1.09218) at rt for 30 min and rinsed with tap water five times. Fluor was added to the wells to create a white background, and the wells were scanned to capture an image. Quantification was performed using ImageJ [13].

#### Calcein retention assay

Du145 (7.5 × 10^3^) and 22Rv1 (10 × 10^3^) cells were seeded on black 96-well plates the day prior to BRPF inhibitor exposure (5 µM, 24 h). Cells were then incubated with 125 nM calcein-AM (Invitrogen™, C1430) for 30 min at 37 °C in the dark. Images were acquired by an inverted fluorescence microscope (Leica DMI8). The BRPF1 inhibitors, namely OF1 (17124), PFI4 (17663), and GSK5959 (18123), were purchased from Cayman.

#### CellTiter-Glo® (CTG) luminescent cell viability assay

Du145 (4 × 10^3^) and 22Rv1 (7.5 × 10^3^) cells were seeded on 96-well plates the day prior to drug exposure. CellTiter-Glo Reagent (Promega, G7570) was added to the wells (1/10) and incubated for 30 min at 37 °C protected from light. Luminescent signal was detected by using a luminometer (Synergy H1 Hybrid reader, BioTek). Cell viability normalization was performed as a percentage with the formula: % Cell Viability = (Treated Cell RLU−blank/Untreated Cell RLU−blank) × 100.

#### Cell cycle analysis

Du145 (3 × 10^4^) and 22Rv1 (6 × 10^4^) cells were seeded on 12-well plates the day prior to treatments. Cells were washed with PBS and fixed for at least 3 h with 70% ethanol (Merck, 100983). Cells were washed with PBS, stained with 200 µL of Cell Cycle Reagent (Luminex Corporation, MCH100106) for 20 min at rt in the dark and analyzed by using the Muse Cell Analyzer with 5000 events.

#### Cell death analysis

Du145-DtxR (2 × 10^5^) cells were seeded on 6-well plates the day prior to drug treatments. Cells were harvested by trypsinization and analyzed for cell death mode using Annexin V & Dead Cell Kit (Luminex Corporation, MCH100105) and Caspase-3/7 Kit (Luminex Corporation, MCH100108). For annexin-v staining, cells were resuspended in 1% FBS containing PBS, and 100 µL of this suspension was mixed with 100 µL of annexin dye (final concentration was 250 cells/μL). The mixture was incubated for 20 min at rt and subsequently analyzed using the Muse Cell Analyzer with 5000 events. For the caspase 3/7 activity assay, 50 μL of cell suspension in 1× Assay Buffer was mixed with 5 μL of the Muse™ caspase-3/7 Reagent working solution (prepared by diluting Muse™ Caspase 3/7 Reagent at a 1:8 ratio in PBS). The mixture was thoroughly mixed, and the loosely capped tubes were incubated for 30 min in a 37 °C incubator with 5% CO_2_. After incubation, 150 μL of Muse™ Caspase 7-AAD working solution was added (prepared by diluting Muse™ Caspase 7-AAD at a 1:75 ratio in 1× Assay Buffer) to each tube, followed by incubation at rt for 5 min, protected from light. Subsequently, the samples were analyzed using the Muse Cell Analyzer with 5000 events.

#### qRt-PCR

Total RNA was extracted with NucleoSpin™ RNA Plus Isolation Kit (Macherey Nagel, 740984.50) and cDNA was synthesized from 1 µg total RNA using the M-MLV Reverse Transcriptase (Thermo Fisher Scientific, 28025013). cDNA (10 ng) was amplified using LightCycler 480 SYBR Green I Master mix (Roche Diagnostics, 04707516001). The reaction mixture (10 μL) contained 0.15 μM specific primers (listed in Sup. Table 1) for target genes and incubated at 95 °C for 5 min, followed by 40 cycles of 95 °C for 10 s, 60 °C for 30 s, and 72 °C for 30 s. β-Actin was used as reference control and qRt-PCR was run on the PikoReal Real-Time PCR System (Thermo Fisher Scientific). The relative fold change in gene expressions were measured with the 2^(−ΔΔCT)^ method.

#### DNA copy numbers by qPCR

To validate the genomic copy number increase of ABCB1, the 7q21.12 region was scanned as stated in [14]. Genomic DNA was extracted from 22Rv1 and Du145 cells using Nucleospin Tissue kit (Macherey Nagel, 740952.50) according to the manufacturer’s protocol. Using genomic DNA as a template (10 ng), the quantification of ABCB1 copy number was performed with LightCycler 480 SYBR Green I Master mix (Roche Diagnostics, 04707516001), using forward and reverse primers (0.15 μM each) covering intron 11 and exon 12 which produce 71 bp of PCR product. The reaction mixture (20 μL) was incubated at 95 °C for 5 min, followed by 40 cycles of 95 °C for 10 s, 55 °C for 30 s, and 72 °C for 30 s. ABCB1 genomic amplification primer sequences are listed in Sup. Table 1.

#### SDS-PAGE and western blotting

Proteins were extracted by RIPA lysis buffer (EcoTech Biotechnology, RIPA-100) containing cOmplete™ protease inhibitor cocktail (Merck, 11697498001), PMSF (Merck, 10837091001) and phosSTOP phosphatase inhibitor (Merck, 4906845001) at 4 °C for 30 min. Protein concentrations were determined using BCA assay (Thermo Fisher Scientific, 23225). The Laemmli sample buffer (Bio-Rad Laboratories, 1610747) and DL-Dithiothreitol (Sigma, D0632) were added to the proteins and boiled at 95 °C for 7 min. Proteins (20–30 µg) were subjected to SDS-PAGE and then transferred to PVDF membranes (Bio-Rad Laboratories, 1620177). The membranes were then blocked with 5% NFDM (Bio-Rad Laboratories, 1706404) at rt for 1 h and blotted with the following primary antibodies at 4 °C overnight: β-Actin (Abcam, ab8224), β-Tubulin (Abcam, ab6046), and MDR1/ABCB1 (Cell Signaling Technology, 13978). The membranes were washed with TBST and incubated with the HRP-conjugated secondary antibodies (Goat pAb to Rabbit and Mouse IgG, Abcam, ab205718, ab205719) for 1 h at rt. Signal was developed using Immobilon Forte Western HRP substrate (Millipore, WBLUF0100) and imaged by LI-COR Odyssey FC Imaging system (LI-COR Biosciences).

#### Cellular Thermal Shift Assay

PCa cells (3.5 × 10^5^) were treated with Dtx (62.5 nM), Cbz (32.5 nM), and BRPF inhibitors (5 µM) for 1 h at 37 °C and 5% CO_2_. Treated and untreated cells were centrifuged, and the pellet was resuspended with PBS containing cOmplete™ protease inhibitor cocktail (Merck, 11697498001) and 1 mM PMSF (Merck, 10837091001). The cell suspension was subsequently divided equally into 0.2 ml tubes, with 100 µL in each, and heated for 3 min at the indicated temperatures (44 °C, 46 °C, 48 °C, 50 °C, 52 °C) using the T100™ Thermal Cycler from Bio-Rad Laboratories. Cells were snap-frozen and thawed twice and centrifuged at 17.000 g for 15 min at 4 °C. The protein-containing supernatant was collected and subjected to SDS-PAGE and western blotting.

#### Histone extraction

Du145 (1 × 10^6^) cells were treated with BRPF inhibitors (5 µM, 1–24 h). Treated and untreated cells were lysed, and histones were extracted, as previously described [15]. The antibodies used were as follows: Acetyl-Histone H3 (Lys9) (Cell Signaling Technologies, 9649); Acetyl-Histone H3 (Lys18) (Cell Signaling Technologies, 13998); Acetyl-Histone H3 (Lys27) (Cell Signaling Technologies, 4353); Histone H3 (Abcam, ab18521) and the HRP-conjugated secondary antibody (Goat pAb to Rabbit, Abcam, ab205718).

#### RNA-sequencing and analysis

Analysis was performed to identify differentially expressed genes; (i) between Du145-DtxR and parental cells, (ii) Du145-CbzR and parental cells, (iii) siBRPF1 and siControl. The comparisons were made with at least 2 biological replicates per treated cell line. Each paired-end sample FASTQ file was assessed for adapter contamination using FastQC. None of the samples required adapter trimming. The sequencing data was aligned using STAR (transcriptome mapping) [16], to the GENCODE GRCH38 [17] Human reference transcriptome. Salmon [18] was used to quantify the STAR transcriptome BAM files based on the transcripts per million method. All samples achieved a mapping rate of greater than 85%, the data was assessed and summarised using MultiQC. In order to aggregate the transcript-level abundance to the gene-level the R package tximport [19] was used. The package DESeq2 [20] was used to analyze the gene-level count data, identifying differentially expressed genes for each of the three contrasts. Significant differentially expressed genes (DEGs) were defined as genes with a log-fold change > 1 (upregulated) or log-fold change < −1 (downregulated) and a false-discovery rate (FDR) adjusted *P*-value < 0.05. The significant results from the gene expression analysis were annotated using the Biomart database [21], to provide descriptions of gene function. Volcano, MA (base-2 log fold-change versus normalized mean counts), and log-fold change comparison plots generated using ggplot2 were used to visualize the gene expression analysis results.

#### ChIP-qPCR

Du145-DtxR cells were crosslinked with %1 formaldehyde (Sigma Aldrich, 818708) at rt for 10 min. Cross-linking was terminated by the addition of glycine (Sigma Aldrich, G7126) (125 mM, 5 min) and cell suspension was centrifuged at 1000 g for 5 min at 4 °C. Pellet was washed with cold PBS twice and resuspended in ChIP Lysis Buffer (50 mM HEPES; 1 mM EDTA; 140 mM NaCl; 1% Triton X-100; 0.1% Na-deoxycholate; 1% SDS; 1 mM PMSF; Protease Inhibitor Cocktail) and incubated on ice for 10 min. The released chromatin was sonicated with Bioruptor Plus sonicator (Diagenode) and was centrifuged at 10.000 g for 10 min at 4 °C. The supernatant containing the sheared chromatin (fragment sizes of 100–500 bp) was collected. The chromatin preparation was incubated with prewashed Dynabeads® (Thermo Fisher protein A magnetic beads, 10001D) at 4 °C for 30 min to avoid non-specific binding (preclearing). BRPF1 antibody (Abcam, ab259840), Anti-HA antibody (Biolegend, 901502) and a non-specific IgG antibody (Sigma Aldrich, PP64B) for negative control were incubated with magnetic beads in PBST for 1 h at rt. The magnetic bead-antibody complex and pre-cleared chromatin preparation was incubated overnight. The magnetic beads were washed with ChIP lysis buffer, low-salt buffer (0.1% SDS, 1% Triton-X 100, 2 mM EDTA, 20 mM Tris-HCl, 140 mM NaCl, pH 8.1), high salt buffer (0.1% SDS, 1% Triton-X 100, 2 mM EDTA, 20 mM Tris-HCl, 500 mM NaCl, pH 8.1), LiCl-containing buffer (0.25 M LiCl, 1% IGEPAL-CA 630, 1% deoxycholic acid, 1 mM EDTA, 10 mM Tris, pH 8.1), Tris-EDTA solution twice for 5 min, respectively. ChIP’ed DNA was eluted using an elution buffer containing 1% SDS (Bio-Rad, 1610418) and 0.1 M NaHCO_3_ (Merck, S6014) and reverse cross linked by incubating at 37 °C with RNase A (0.3 mg/ml) (Thermo Fisher Scientific, 12091039) and NaCl (Merck, S9888) (5 M) for 30 min, followed by addition of proteinase K (0.3 mg/ml) (Thermo Fisher Scientific, EO0492) at 65 °C for 1 h. DNA was purified by using the ChIP DNA Clean & Concentrator kit (Zymo Research, D5205). Primers were designed by using UCSC Genome (https://genome.ucsc.edu/) and Ensembl Genome Browser (https://www.ensembl.org/index.html) and primer sequences are listed in Sup. Table 1. For the quantitative and simultaneous analysis of immunoprecipitated DNA, qPCR was run on the LightCycler 480 (Roche). ChIP-qPCR results were calculated by % input method. To exogenously pull down BRPF1, BRPF1 was cloned with an HA tag. BRPF1 cDNA sequence was amplified from the GFP-BRPF1 plasmid (Addgene: 65382) and ligated into the lentiviral expression vector pLEX-305-C-dTAG (Addgene: 91798).

#### Analysis of clinical data

Pan-cancer analysis of whole genomes [22], The Cancer Genome Atlas Prostate Adenocarcinoma (TCGA-PRAD) [23] and Firehose Legacy, Fred Hutchinson CRC [24], Metastatic Prostate Adenocarcinoma (SU2C/PCF Dream Team, [25, 26]), and Metastatic Prostate Adenocarcinoma (MCTP) [27] datasets were accessed via cBioPortal [28, 29]. Statistical analysis using the unpaired t-test was conducted with Prism 8 (GraphPad Software, Inc.).

### Statistical analysis

All data were analyzed in Prism 8 (GraphPad Software, Inc.) and statistical tests were applied as described in the figure legends. Combination index (CI) values were calculated using CalcuSyn software (Biosoft) based on the method described by Chou and Talalay to assess the synergism [30].

## Results

### Establishment and characterization of Dtx and Cbz resistant PCa cells

Dtx and Cbz resistant cell lines (DtxR and CbzR, respectively) were generated from CR-PCa cell lines, Du145 and 22Rv1. The parental cells were allowed to grow for 24 h before being treated (72 h) with dose escalation (Sup. Figure 1A). Cell growth assays were performed to assess the acquired resistance, revealing a significant number of viable cells in the resistant compartments following taxane exposure (Sup. Figure 1B, C). The IC_50_ values of resistant cells were significantly higher than those of parental cells, exhibiting ~40–560 fold increase for DtxR and ~14–170 fold for CbzR (Table 1). We showed that these viability differences observed in cells were indeed associated with cell death, as evident by the significant increases in annexin-v and caspase 3/7 positive cells in parental cells (Sup. Fig. 1D). Even at higher doses, resistant cells did not display these apoptosis markers, thus reflecting the non-responder state in accordance with the viability assays. Next, we assessed the target engagement (TE) for tubulin, the primary target of taxanes, using Cellular Thermal Shift Assay (CETSA), which measures TE in intact cells by heating them and determining if taxanes stabilize the tubulin against thermal denaturation. As shown in Sup. Fig. 1E, a higher level of target engagement was observed in parental cells, indicating their ability to retain the drug compared to resistant cells. These findings suggest a potential inadequacy of taxane uptake in resistant cells, leading to a consequent lack of target engagement. In line with our findings, a study involving both sensitive and resistant cells, CETSA TE measurements correlated with taxane sensitivity and efficiently revealed the presence of acquired drug resistance [31].

**Table 1:** IC_50_ values and relative resistance of Dtx/Cbz resistant Du145 and 22Rv1 cells compared to parental cells.

Cell line	IC_50_ to Dtx (nM)	Relative resistance
Du145-P	0.45	1
Du145-DtxR	>250	>555.56
22Rv1-P	3.05	1
22Rv1-DtxR	>125	>40.98

Cell line	IC_50_ to Cbz (nM)	Relative resistance
Du145-P	1.48	1
Du145-CbzR	>250	>168.92
22Rv1-P	5.20	1
22Rv1-CbzR	72.98	14.03

Cells were incubated with increasing concentrations of Dtx/Cbz for 72 h prior to the CTG assay.

Interestingly, while the cells were cross-resistant against other chemotherapeutics such as paclitaxel and doxorubicin (Sup. Fig. 2A), they became more susceptible to platinum group drugs (Sup. Fig. 2B), suggesting that a common mechanism acted to detoxify drugs that are substrates of drug efflux pumps, at the expense of becoming more vulnerable towards other drugs. All these data have brought us to a point where we can elucidate the mechanism underlying taxane resistance in our well-characterized PCa cell models.

### Transcriptome profiles of taxane-sensitive and -resistant PCa cells

**Fig. 1:**
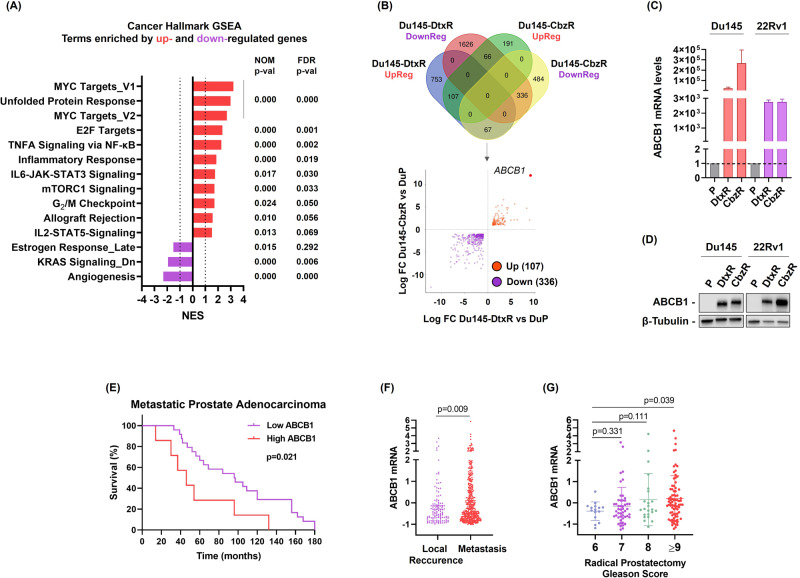
Upregulation of ABCB1 in taxane-resistant cells and its clinical relevance in advanced PCa. **A** Gene set enrichment (GSEA) analysis using hallmark gene sets from the MSigDB revealed the upregulation of genes involved in MYC signaling, unfolded protein response (UPR), NF-κB and E2F signaling (FDR < 0.05, and Log_2_FC ≥ 0.5 or ≤ −0.5). GSEA was performed using the h.all.v2023.1.Hs.symbols.gmt dataset in the MsigDB database. **B** DEGs (FDR < 0.05, and Log_2_FC ≥ 1 or ≤−1) from resistant cells were further distributed based on their expression changes in the same direction (up or down). The red and purple scatters indicate up- and downregulated DEGs, respectively, in resistant Du145 cells. **C** Expression levels of ABCB1 mRNA were determined by qRt-PCR. Data is the mean ± SEM. **D** ABCB1 protein expression levels in parental and taxane resistant PCa cells were determined by western blotting. **E** Comparison of patient survival based on ABCB1 expression levels using a Kaplan-Meier plot (Metastatic Prostate Adenocarcinoma, MCTP). **F** ABCB1 mRNA levels in clinical specimens (TCGA-PRAD) were investigated by using cBioPortal and its relationship with the occurrence of local recurrence and metastasis (Pan-cancer Analysis of Advanced and Metastatic Tumors, BCGSC); (**G**) and gleason score (Metastatic Prostate Adenocarcinoma, SU2C/PCF Dream Team) were shown as scatter plots. Uncropped western blot images corresponding to Fig. 1D were shown in Supplemental Material.

We conducted a whole transcriptome analysis between taxane-sensitive and -resistant Du145 cells to unravel potential molecular mechanisms of resistance. Among the differentially expressed genes (DEGs), the top 10 upregulated and downregulated genes in Du145-DtxR and Du145-CbzR cells are listed in Sup. Tables 2 and 3, respectively. The transcriptomic profile was further analyzed using gene set enrichment analysis (GSEA). The ranked gene sets were tested on the hallmark gene sets, which represent well-defined biologic states and processes (MSigDB, Broad Institute). The relative normalized enrichment scores (NES) demonstrated that MYC signaling, unfolded protein response (UPR), E2F and NF-κB signaling were the most significantly enriched gene sets in Du145-DtxR cells (Fig. 1A). The genes demonstrating core enrichment within these gene sets are listed in Sup. Table 4.

The enrichments of “MYC Targets_V1” and “E2F Targets” were also observed in Du145-CbzR cells, similar to Dtx-resistant cells. This suggests a potential involvement of MYC and E2F signaling pathways in mediating resistance mechanisms across different taxanes (Sup. Fig. 3). However, there is also some degree of variation in the underlying mechanisms within each cell. Therefore, we utilized a Venn diagram to identify the common gene pool specific to taxane resistance in both cells, categorizing the DEGs based on their expression changes (up or down) (Fig. 1B). Our analysis revealed that 107 genes were commonly upregulated and 336 were downregulated in taxane resistant cells. Notably, ABCB1 emerged as the gene with the highest transcriptional change in both resistant cells (Fig. 1B).

### ABCB1 overexpression in taxane-resistant PCa cells

The top transcriptional change took place for the ABCB1 gene, with a 10.3 and 12.9-fold (log_2_ scale) upregulation in Du145-DtxR and Du145-CbzR, respectively, compared to parental cells. After validating the transcript expression of ABCB1 (Fig. 1C), we confirmed the corresponding protein upregulation in all taxane-resistant cells (Fig. 1D). The patient data analysis highlighted the clinical significance of ABCB1 expression, with ABCB1-high patients showing unfavorable outcomes in terms of overall survival (Fig. 1E). Furthermore, the analysis of advanced and metastatic tumors revealed a significant relationship between higher ABCB1 mRNA levels and an increased incidence of metastasis compared to patients with local recurrence (Fig. 1F). In another dataset of patients with metastatic disease, an association was observed between higher Gleason scores and increased ABCB1 mRNA expression (Fig. 1G). Taken together, these findings provide evidence supporting a potential relationship between ABCB1 expression and disease progression.

**Fig. 2:**
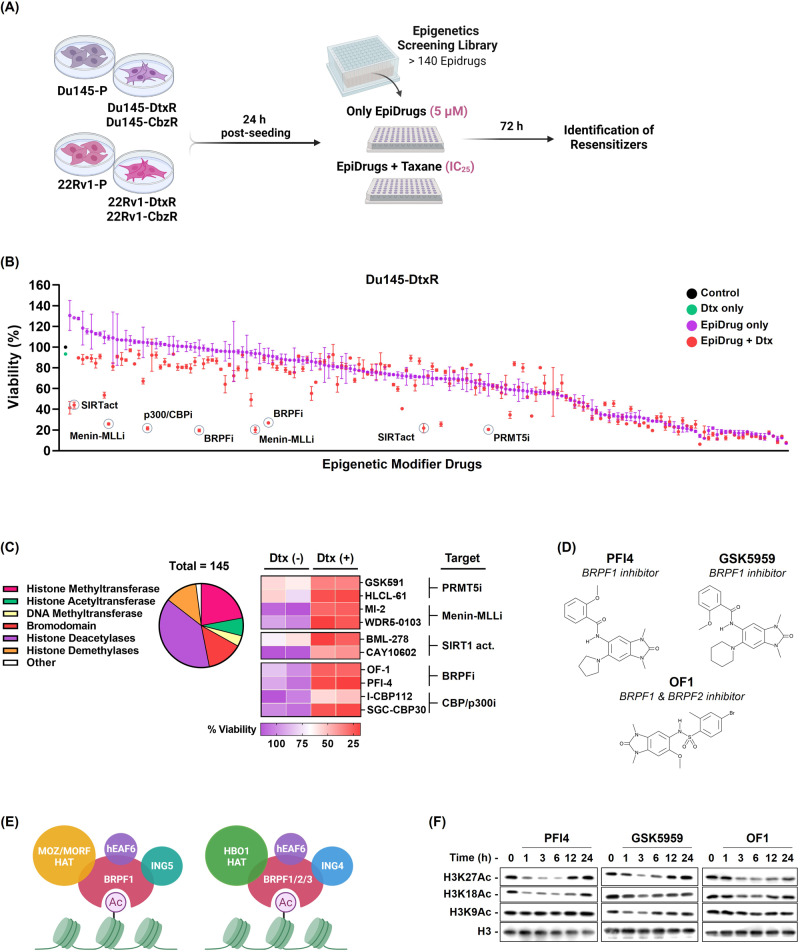
Epigenetic drug screening reveals vulnerabilities for taxane-resistant PCa cells. **A** Schematic view of the epidrug library screening. The figure was generated using BioRender software. **B** Screening of 145 targeted drugs to identify epigenetic modulators capable of overcoming taxane resistance was performed using the SRB assay and the result obtained in Du145-DtxR cells is shown representatively. Purple dots show the cell viabilities upon the standalone use of each epidrugs, while the red dots indicate the cell-killing effect of their combination with Dtx. Re-sensitizers ranked in the top 5 groups are highlighted in circles. **C** Pie Chart illustrates the epigenetic proteins targeted by the small molecules (epidrugs) in the library. Epidrugs were screened for resistance reverter activity which resulted in the identification of 5 classes of epigenetic targets (PRMT5, Menin-MLL, SIRT1, BRPF, and CBP/p300) that induced the most pronounced resensitization when applied to resistant cells. **D** Molecular structure of BRPF inhibitors. **E** Cartoon representation of the BRPF family and (**F**) demonstration of the efficacy of BRPF inhibition on histone acetylation by western blotting. Uncropped western blot images corresponding to **F** were shown in Supplemental Material.

One of the potential causes for the increased expression of ABCB1 is gene amplification. Therefore, we examined ABCB1 gene copy numbers in all PCa cell lines. ABCB1 amplification was evident only in Du145-CbzR cells (Sup. Figure 4). The reason for such upregulation of ABCB1 could also be via promoter activation, indicating possible epigenetic regulations. The absence of amplification in 3 out of 4 cells has led us to consider the possibility of epigenetic regulation, which also drove us to screen these cells with an epidrug library to reveal their vulnerabilities (Fig. 2).

To assess the importance of ABCB1 expression in taxane resistance, we used siRNA to target ABCB1 in resistant Du145 cells (Sup. Fig. 5A, B). However, we encountered difficulties in achieving effective knockdown of ABCB1 using the same siRNA in resistant 22Rv1 cells. Therefore, we proceeded to knock out ABCB1 expression in these cells (Sup. Fig. 5C, D). The inhibition of ABCB1 expression in all resistant cells resulted in decreased survival at the doses of taxanes that showed minimal cytotoxicity in the previous results, highlighting the significance of ABCB1 expression in mediating taxane resistance (Sup. Fig. 5E–H). Additionally, the cells were treated with epothilone B (Epo B), a tubulin targeting drug which is not an ABCB1 substrate. Resistant cells exhibited similar viability curves to parental cells upon Epo B exposure, providing further evidence that the observed difference between the resistant and parental cells was predominantly dependent on ABCB1 (Sup. Fig. 5I, J).

### Episensitization of taxane-resistant PCa cells

Targeting epigenetic modulators in PCa holds promise with numerous pre-clinical studies supporting its potential. For instance, Asangani et al. demonstrated that the combination of BET inhibitors with AR antagonists can overcome resistance mechanisms in metastatic CR-PCa models [32]. Xu et al. observed that chemotherapy-resistant PCa cells displayed elevated levels of acyl-CoA and acetylated proteins, making them more sensitive to HDAC inhibition (TSA/SAHA) compared to their parental counterparts [33]. Gupta et al. showed that LSD1 inhibition with HCI-2509 enhanced the response of Dtx-resistant PCa cells to the treatment, leading to a reduction in c-MYC levels [34]. Not surprisingly, with all these studies and accumulated knowledge, the FDA has approved eight small molecules targeting DNA methylation, histone methylation, and histone deacetylation for cancer treatment based on their effectiveness in various solid and hematologic malignancies [35]. Leveraging this trend and the established effectiveness of epidrugs, we performed an epigenetic drug library screen to uncover potential targets and vulnerabilities in taxane-resistant PCa cells (Fig. 2A). The epigenetic drug library has 145 compounds including a majority of currently available agonists and antagonists of epigenetic regulatory enzymes. The top 5 classes of epigenetic targets that induced the most pronounced resensitization when applied to resistant cells were identified as CBP/p300, BRPF, Menin-MLL, PRMT5, and SIRT1 (Fig. 2B, C). The efficacy of the epigenetic drugs was further validated using cell viability assays at different concentrations, revealing a restoration in taxane susceptibility with increasing doses of epidrugs (Sup. Fig. 6). However, subsequent investigation showed that expressing SIRT1 did not have a significant impact on resensitization of taxane-resistant cells. (Sup. Fig. 7A, B). Additionally, silencing CBP, a well-known transcriptional coactivator with histone acetyltransferase activity, resulted in a complete loss of colony-forming ability of cells, indicating their essentiality for PCa cell survival (Sup. Fig. 7C). On the other hand, our laboratory has ongoing studies on Menin-MLL and PRMT5, which are being investigated in detail and will be reported separately. Therefore, the findings from our epigenetic drug library screen led us to prioritize BRPF proteins as a potential target for overcoming taxane resistance in PCa. We also identified a knowledge gap regarding the functions of BRPF epiregulators and the efficacy of BRPF inhibitors (Fig. 2D) in cancer drug resistance, which drove us to highlight these proteins as promising and novel targetable epiregulators in this context.

### Targeting BRPF epiregulators in taxane-resistant PCa cells

The BRPF epigenetic reader family (BRPF1, BRPF2, and BRPF3) acts as scaffolds for assembling HAT complexes of MOZ/MORF and HBO1 families with ING5 and Eaf6, carrying these to chromatin via its bromodomain [36] (Fig. 2E). Both BRPF2 and BRPF3, despite their high sequence similarity to BRPF1, have a preference for forming complexes with HBO1 instead of MOZ and MORF, suggesting a distinct functional behavior of BRPF1. Moreover, BRPF1 directs histone acetylation towards H3 instead of H4, indicating its pivotal role in controlling the substrate specificity of HBO1 [37]. In fact, we observed that treatments with BRPF inhibitors altered the global H3K18, H3K27, and, to some extent, H3K9 acetylation levels, providing evidence of the intracellular activities of these compounds (Fig. 2F). BRPF1 is known to be indispensable for embryonic development [38, 39]; however, its involvement in cancer, with the exception of limited studies, remains poorly characterized.

**Fig. 3:**
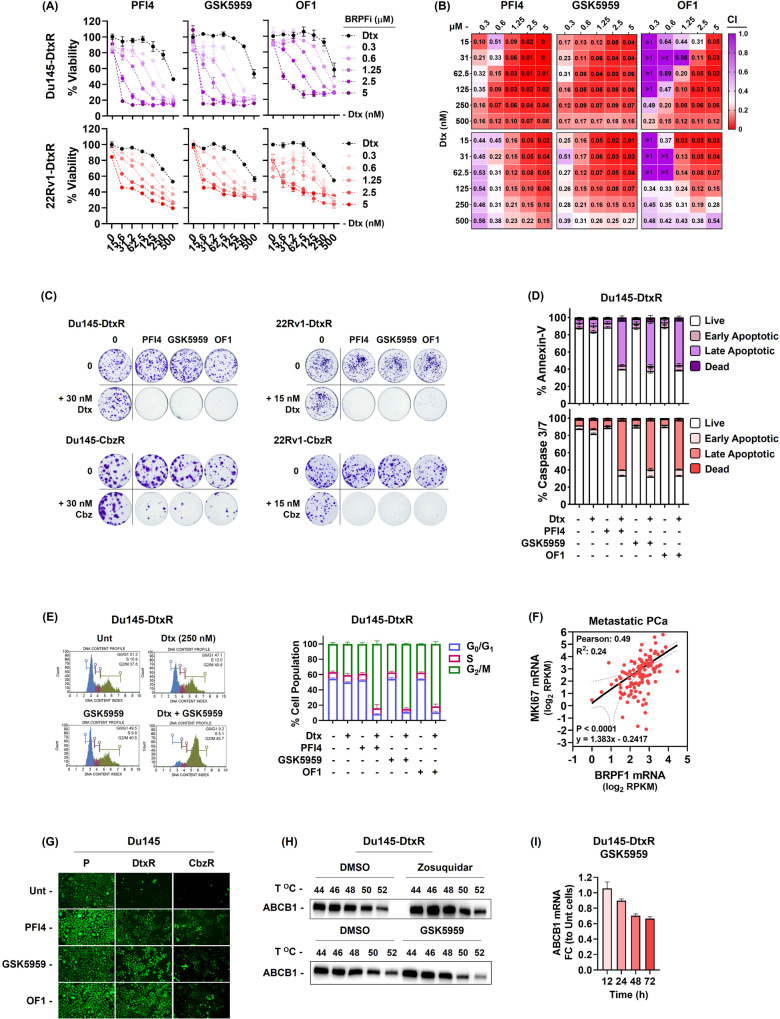
Restoration of taxane susceptibility by BRPF Inhibition. **A** Validation dose-response curves of BRPF inhibitors (PFI4, GSK5959 and OF1) on Dtx-resistant cells. Cells were co-treated with indicated drugs (Dtx; 1–250 nM and BRPF inhibitors; 1.25–5 µM) and the results were obtained by SRB viability assay (72 h). The data is expressed as mean ± SEM. **B** Heat map representation of the Combination Index (CI) values, with red color indicating a synergistic effect. CI was calculated using the CalcuSyn software. **C** Clonogenic images were obtained by treating cells with indicated drugs for 72 h and the colony formation ability was analyzed 10–15 days after drug exposure. **D** Flow cytometry analysis of cell death (48 h) and (**E**) cell cycle distribution (24 h) in resensitized Du145-DtxR cells. **F** The expression of BRPF1 showed a positive correlation with MKi67 expression in metastatic PCa. **G** Calcein retention assay was performed in the absence or presence of BRPF inhibitors (5 µM, 24 h). **H** CETSA for in-cell ABCB1 engagement. Western blots showing thermostable ABCB1 following indicated heat shocks (44 °C, 46 °C, 48 °C, 50 °C and 52 °C) in the presence of Zosuquidar (5 µM) and GSK5959 (5 µM) in Du145-DtxR cells. **I** The expression of ABCB1 was evaluated by qRt-PCR at the indicated time points following treatment with GSK5959 (5 µM). P parental, R resistant. Uncropped western blot images corresponding to **H** were shown in Supplemental Material.

We validated these hits by screening each BRPF inhibitor in a wider dose range and confirmed the resensitizing effect as resistant cells became more responsive to taxanes with increasing doses of these inhibitors (Fig. 3A and Sup. Fig. 8A). Additionally, the observed effect was found to be synergistic, as evident by combination index (CI) values (Fig. 3B). These findings were further supported by colony formation assays, where individual use of either of the drugs had minimal to negligible impact. However, when the two drugs were combined, they effectively eliminated most of the cells (Fig. 3C). Interestingly, parental cells did not show any increased sensitivity to epidrug treatments indicating that BRPF inhibitors have the potential to selectively target resistant cells (Sup. Fig. 8B). To confirm whether the growth inhibition observed in response to the drug combinations was due to cell death, we assessed apoptotic markers. Combining taxanes and epidrugs resulted in increased annexin-v staining and caspase 3/7 activation compared to using either drug alone (Fig. 3D). Furthermore, the G_2_/M arrest of resistant PCa cells by BRPF inhibitors provides further support for the restoration of taxane sensitivity (Fig. 3E and Sup. Fig. 8C). Lastly, increased BRPF1 expression was found to be associated with elevated expression of the cell growth indicator, mKI67, in patients with metastatic PCa, providing evidence for its role in promoting cellular proliferation pathways (Fig. 3F).

Based on the apparent role of ABCB1 in taxane response (Sup. Figure 5), it is reasonable to speculate that these inhibitors could interfere with its function. To test this, we performed calcein efflux assay with BRPF inhibitors. Despite reduced calcein accumulation in resistant cells due to high ABCB1 expression, green fluorescence significantly increased with all BRPF inhibitors, supporting their potential role in inhibiting ABCB1 function (Fig. 3G, Sup. Figure 8D). It could be assumed that these epidrugs can be effective by binding to ABCB1. To investigate the potential binding of BRPF inhibitors with ABCB1, we performed CETSA against ABCB1. Zosuquidar, a known inhibitor of ABCB1, was used as a positive control and not surprisingly, it showed an ABCB1-binding compared to DMSO (Fig. 3H). On the other hand, BRPF inhibitors did not cause any thermal shift, therefore we concluded as they do not engage with ABCB1 physically (Fig. 3H, Sup. Figure 8E). The restoration of taxane sensitivity in resistant cells might be attributed to downstream mechanisms rather than direct binding to ABCB1. Indeed, a time-dependent decrease in the expression of ABCB1 was observed upon treatment with GSK5959 (Fig. 3I). Additionally, the lack of cytotoxic effects observed in RPE-1 cells upon treatment with BRPF inhibitors provides promising evidence for their potential therapeutic application (Sup. Figure 8F). The differential response to epidrug treatments between sensitive and resistant cells can be attributed to the expression of ABCB1. Indeed, knocking out ABCB1 in the resistant cells abolished the effectiveness of BRPF inhibitors, suggesting that the effect of BRPF inhibitors is dependent on the presence of ABCB1 (Sup. Fig. 9).

In order to investigate whether the effect of BRPFi can be recapitulated using gene targeting, we employed both CRISPR-guided knockout and siRNA-mediated silencing strategies. Several attempts to generate BRPF knock out cells failed, as resistant cells did not survive following introduction of targeted gRNAs indicating that the activity of BRPF1 gene became essential for the resistance phenotype. On the other hand, short-term depletion, as in the case of RNA interference, was applicable. The colony formation assay showed that knockdown of BRPF1 suppressed the ability to form colonies (Sup. Figure 10A). However, this effect was observed to be more intense in resistant cells, once again showing the essentiality of BRPF1 in resistant cells. Indeed, stable and specific knock-down of BRPF1 led to a higher number of apoptotic cells in Dtx-resistant cells (Sup. Fig. 10B, C).

**Fig. 4:**
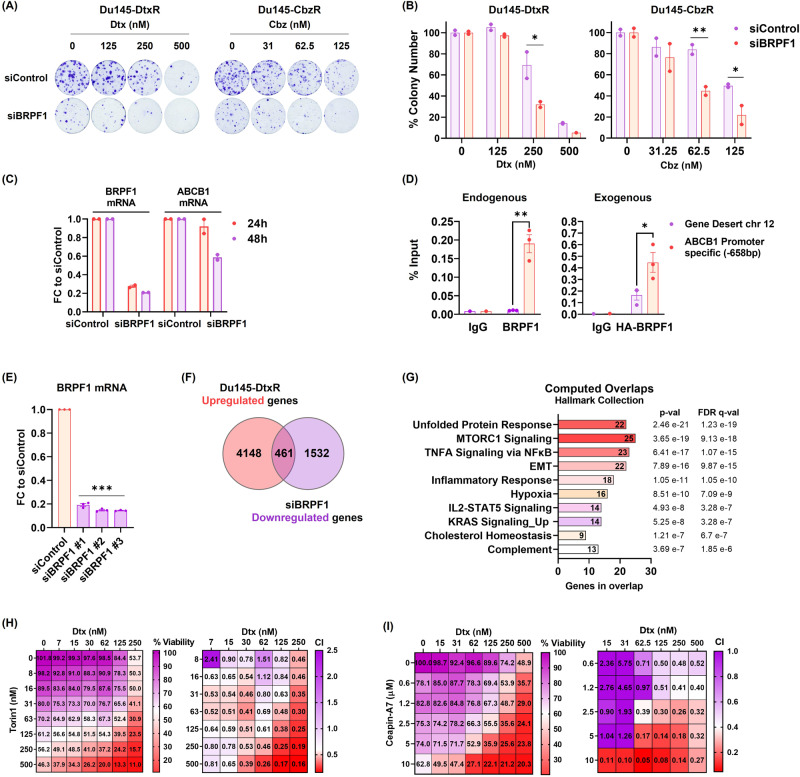
BRPF1 regulates ABCB1 expression and interferes with multiple signalings in resistant cells. **A** Taxane response of siBRPF1 treated resistant cells evaluated by colony formation assay. Representative images and (**B**) quantifications are shown. **C** The expression of ABCB1 was evaluated by qRt-PCR at the indicated time points following treatment with siBRPF1 in Du145-DtxR cells. **D** ChIP-qPCR showing BRPF1 enrichment at the *ABCB1* promoter in Du145-DtxR cells expressing endogenous BRPF1 (left panel) and exogenous HA-tagged BRPF1 (right panel). BRPF1 enrichment at a control region (*Chr12 gene desert*) is also shown. Data are shown as percentage of ChIP input; dots represent individual biological replicates; bars represent mean replicates. **E** BRPF1 mRNA levels of RNA-seq samples from siControl and siBRPF1 in Du145-DtxR cells. **F** Venn diagram showing the number of genes (intersection, 461) whose expression decreased after silencing of BRPF1 among genes with increased expression in Du145-DtxR cells (vs Du145-P). **G** Computed overlaps of the 461 genes in the Hallmark Collection of GSEA (MSigDB) database. **H** The efficacy of Torin1 (mTORC1/2 inhibitor, 8–500 nM) on Du145-DtxR cells was determined by CTG assay and represented as a heat map. **I** The efficacy of Ceapin-A7 (ATF6α inhibitor, 0.6–10 µM) on Du145-DtxR cells was determined by SRB assay and represented as a heat map. The Combination Index (CI) was calculated using the CalcuSyn program. Statistical significance denoted as *p < 0.05, **p < 0.01, and ***p < 0.0001.

While not as pronounced as the effect seen with pharmacological inhibition, knockdown of BRPF1 also led to the resensitization of cells to taxanes (Fig. 4A, B). We also noted a decrease in ABCB1 expression upon BRPF1 knockdown (Fig. 4C). The observed decrease in ABCB1 expression with both pharmacological and genomic suppression implies a potential role of BRPF1 in transcriptional regulation. In order to test whether BRPF1 directly regulates ABCB1 expression, we analyzed the enrichment of BRPF1 on the promoter of ABCB1 in Du145-DtxR cells using ChIP assay. We found that, in comparison with IgG and gene desert region controls, BRPF1 was indeed enriched at the promoter region of ABCB1 (Fig. 4D). To eliminate potential off-target effects of the endogenous BRPF1 antibody, HA-ChIP was performed using exogenous expression of BRPF1, once again showing enrichment at the ABCB1 promoter, further supporting BRPF1-mediated regulation of the ABCB1 gene.

To understand the molecular mechanisms behind BRPF1-mediated reversion of drug resistance, we wanted to examine the transcriptome of siBRPF1 treated Dtx-resistant cells (Fig. 4E). We limited the gene pool of interest by filtering the upregulated genes whose expression was previously determined in resistant cells (relative to parental cells) but showed an opposite direction of regulation after siBRPF1 treatment (Fig. 4F and Sup. Fig. 11). Among the pathways/signal transductions in which a total of 461 genes overlaps in the GSEA (MSigDB) database (Hallmark Collection), mTORC1 and Unfolded Protein Response (UPR) have emerged as the most significant overlaps (Fig. 4G and Sup. Table 5). Torin1 (an inhibitor of mTORC1/2) and Ceapin-A7 (an inhibitor of ATF6α, which is one of three main UPR sensors) were used to assess the essentiality of these pathways in reversing resistance. Administration of the inhibitors restored the sensitivity of resistant cells to Dtx, while having no effect on the response of sensitive cells, mimicking the effect observed with BRPF inhibitors (Fig. 4H, I and Sup. Fig. 12). These observations suggest that BRPF1 may exert a regulatory role in modulating ABCB1 expression through its interaction with the UPR and mTORC1 signaling pathways. Indeed, the combination of ABCB1 silencing and mTOR inhibition enhances the sensitization of cells to taxanes, suggesting a mechanism involving multiple downstream pathways regulated by BRPF1 (Sup. Fig. 13). These results indicate the involvement of diverse mechanisms in overcoming taxane resistance. The combined targeting of ABCB1 and mTOR pathways may act through complementary mechanisms, such as inhibiting drug efflux and modulating intracellular signaling cascades, ultimately leading to the restoration of drug sensitivity.

Upon examining the functional clusters of upregulated genes with siBRPF1 treatment, it was reasonable to observe the emergence of gene sets related to PCa, such as “androgen response” or “mitotic spindle” and “G_2_M checkpoint”, given the mechanism of action of taxanes (Sup. Fig. 11). Comprehensive studies are needed to investigate the significance of these genes in taxane resistance (Sup. Table 6).

**Fig. 5:**
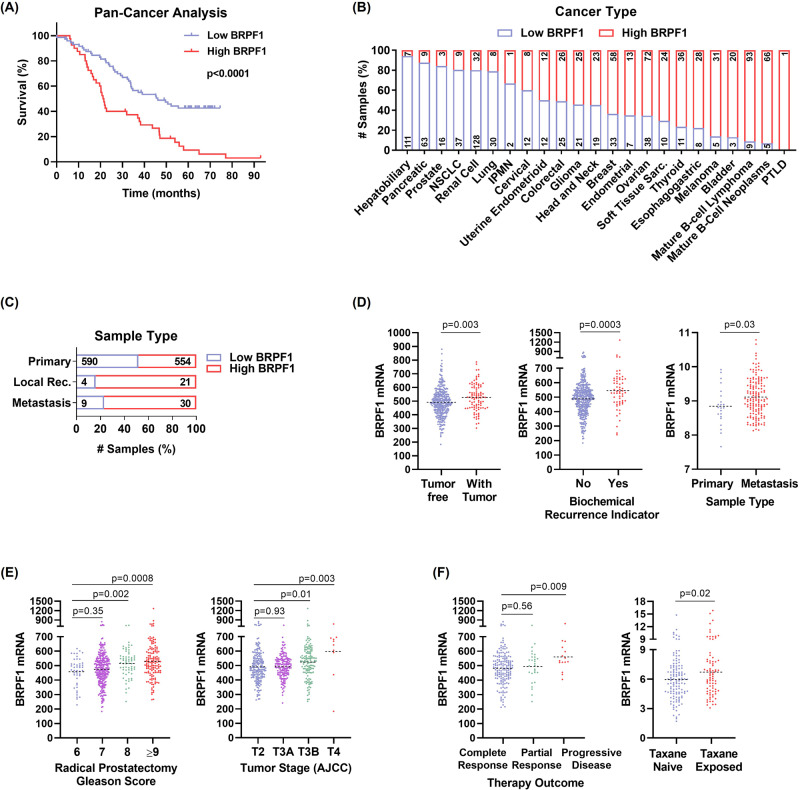
Clinical significance of BRPF1 expression. **A** BRPF1 mRNA levels were analyzed in the Pan-Cancer Analysis dataset (ICGC/TCGA, Nature 2020) from cBioPortal. Kaplan-Meier plot showing the comparison of patient survival based on BRPF1 expression levels. The patients were classified into Low BRPF1 and High BRPF1 groups based on the expression median. The distribution of BRPF1 expression (**B**) across different cancers and (**C**) sample types. The numbers within the column bars correspond to the sample size. **D** BRPF1 mRNA levels in clinical PCa specimens were examined for their correlation with neoplasm status (Prostate Adenocarcinoma, TCGA, PanCancer Atlas), recurrence (Prostate Adenocarcinoma, TCGA, Firehose Legacy), sample type (Prostate Adenocarcinoma, Fred Hutchinson CRC, Nat Med 2016) (**E**) Gleason score (Prostate Adenocarcinoma, TCGA, Firehose Legacy), tumor stage (Prostate Adenocarcinoma, TCGA, Firehose Legacy), (**F**) therapy outcome (Prostate Adenocarcinoma, TCGA, Firehose Legacy), and taxane exposure status (Metastatic Prostate Adenocarcinoma, SU2C/PCF Dream Team, PNAS 2019). The results were presented as scatter plots.

We analyzed publicly available clinical data and initially performed a pan-cancer analysis. We divided the patients into low and high BRPF1 expression groups using the median values determined by the cBioPortal (Fig. 5). Our findings demonstrated a significant decrease in the overall survival of patients exhibiting high levels of BRPF1 expression (Fig. 5A). Further analysis of the specific cancer types revealed higher BRPF1 expression in lymphoma, while lower expression levels were observed in hepatobiliary, pancreatic, and prostate cancers (Fig. 5B). In the analysis of sample types, we observed a higher expression of BRPF1 in local recurrence and metastasis (Fig. 5C). This finding was further supported by the increased BRPF1 levels observed in patients with tumors compared to those without tumors (Fig. 5D). Moreover, BRPF1 expression might be a potential recurrence indicator, as higher expression was observed in patients upon recurrence (Fig. 5D). Supportingly, its expression was found to be elevated in metastatic samples (Fig. 5D). These findings suggest that despite being categorized in the low BRPF1 group, the expression of BRPF1 in PCa may be implicated in disease progression. Indeed, we found a significant association between BRPF1 expression levels and PCa progression, with higher expression levels being correlated with increasing Gleason score and tumor stage (Fig. 5E). BRPF1 expression also seems to reflect therapy outcome, as patients with higher expression levels tend to have a poorer response to treatment and more progressive disease (Fig. 5F). Additionally, it is noteworthy that an increase in BRPF1 expression was observed in patients who were exposed to taxane (Fig. 5F), further emphasizing its potential relevance in therapy outcome and disease progression. Overall, these data highlight the clinical significance of BRPF1 expression as a potential biomarker for PCa progression.

The analysis of BRPF gene expression in our isogenic cells indicated minimal changes overall, with variations observed among PCa cells and taxanes (Sup. Fig. 14A). The lack of observed increase in BRPF1 expression in our cells, unlike in patient tissues, could potentially be attributed to the developed drug resistance that has become chronic in cells. Nevertheless, there could be differences in BRPF activities between resistant and parental cells, which warrants further investigation. If this was indeed associated with a chronic state, our hypothesis was that PCa parental cells may acutely increase BRPF expression following taxane exposure, as supported by the observation of BRPF1 upregulation in PCa patients (Fig. 5F). Indeed, the treatment with Dtx resulted in a significant increase in the expression of BRPF1 (Sup. Fig. 14B). In addition, we also observed an upregulation of ABCB1 expression following Dtx treatment (Sup. Fig. 14B).

**Fig. 6:**
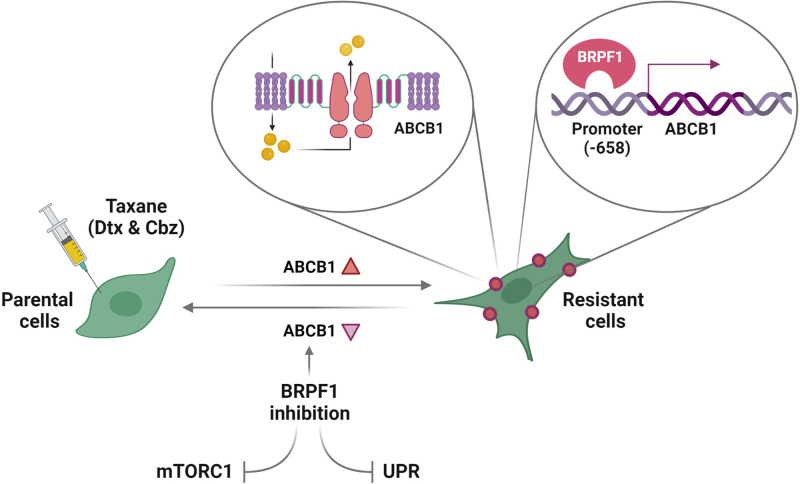
Graphical abstract. The figure illustrates the major findings of the study.

Our findings provide the first evidence for (i) the potential of BRPF inhibitors to overcome taxane resistance and (ii) the regulatory role of BRPF1 through direct modulation of ABCB1 expression at the promoter level. Additionally, we showed that BRPF1 may indirectly regulate ABCB1 expression by interfering with UPR and mTORC1 signaling pathways. The graphical abstract summarizing the study is depicted in Fig. 6.

## Discussion

In this study, we searched for novel epigenetic targets for taxane-resistant CR-PCa treatment. A set of Dtx and Cbz resistant cell lines were derived by pulse selection (Sup. Fig. 1A). The cells became less responsive to taxanes, exhibited increased IC_50_ values (Table 1) and survival (Sup. Fig. 1B, C) and less apoptosis (Sup. Fig. 1D). Not surprisingly, we observed less microtubule engagement of taxanes in drug-resistant cells (Sup. Fig. 1E), which can be a result of; i. acquired mechanisms that prevent drug accumulation and thus prevent target engagement or ii. tubulin mutations. To elucidate the possible mechanisms falling into the first group, we performed RNA-seq analysis (Fig. 1A, B) and detected ABCB1 upregulation in all taxane resistant cells (Fig. 1C, D). Inhibition of ABCB1 expression by using siRNA or gRNA resensitized the resistant cells (Sup. Fig. 5A–H). Furthermore, by using Epothilone B we showed that the observed difference between the resistant and parental cell lines was ABCB1-dependent (Sup. Fig. 5I, J). The upregulation of ABCB1 emerges as a frequent and common mechanism underlying Dtx or Cbz resistance in PCa, as highlighted by previous studies characterizing taxane-resistant PCa cells generated through a similar approach to ours [40–45]. Only one of the resistant lines (Du145-CbzR) exhibited copy number amplification (Sup. Fig. 4). Lombard et al. demonstrated the activation of the ABCB1 amplicon in taxane-resistant PCa cells and proposed its involvement in resistance beyond ABCB1 alone [43]. Their RNA-seq analysis revealed coordinated overexpression of ABCB1-amplicon genes. Among them, targeting the highly overexpressed gene RUNDC3B, in addition to ABCB1, resulted in reduced cell viability and enhanced sensitivity of cells to taxane. This study, along with others as reviewed by Genovese et al., has demonstrated that there is an overexpression or amplification of the genomic region (7q21.12) in taxane-resistant cells [46]. Our analysis of the RNA-seq data revealed significant upregulation in 5 out of 11 genes within the ABCB1 amplicon in Du145-CbzR cells, including HNRNPA1P9 (Log_2_FC: 6.7), ABCB4 (Log_2_FC: 5.9), CROT (Log_2_FC: 2.6), TP53TG1 (Log_2_FC: 2.5), and TMEM243 (Log_2_FC: 2.49). Some of these genes were also among the top 10 upregulated genes in Cbz-resistant cells, as shown in Supp. Table 3. However, in Du145-DtxR cells, only 2 of the 11 genes were upregulated:HNRNPA1P9 (Log_2_FC: 3.33) and RUNDC3B (Log_2_FC: 1.18), indicating the presence of mechanisms other than gene dosage, such as epigenetic events, in driving the overexpression of ABCB1.

Although several studies have demonstrated the molecular profiling of taxane resistance [47–52], epigenetic modifiers have not been elucidated in taxane resistant CR-PCa. In an attempt to uncover the epigenetic modifiers (Fig. 2A, B) an epi-drug screen was performed and 5 major classes of targeted molecules (CBP/p300, Menin-MLL, SIRT, PRMT5, and BRPF) appeared as hits with reversion capacity (Fig. 2C). To our knowledge, this study is the first to report these enzymes as targets and the potential use of their inhibitors in taxane resistant cells (Fig. 2D and Sup. Fig. 6). Targeting BRPFs in resistant cells resulted in resensitization, as evident by CI values of synergy and cell death analyses (Fig. 3A–D). Indeed, cells were able to enter G_2_/M arrest upon BRPF inhibition (Fig. 3E). These findings showed that, in ABCB1 upregulated cells, taxanes could act in a cellular environment where BRPFs were inhibited and therefore highlight an important role for BRPFs as determinants of taxane susceptibility. Previous studies indicated a role for BRPF1 in bone maintenance [36], development of vertebrates ([53]), mouse embryo [38], forebrain [39] and fetal hematopoietic stem cells [54], as well as in learning and memory [55] and causing neurodevelopmental disorders in humans when mutated [56]. Moreover, somatic BRPF1 mutations have been identified in sonic hedgehog medulloblastoma [57] and pediatric cancers [58]. Although there are not many studies on the function of BRPFs in cancer, its significance has been noted in liver cancer [59], lower-grade gliomas [60] and PCa [61]. Gene ablation or pharmacological inactivation of BRPF1 significantly attenuated HCC cell growth in vitro and in vivo [59]. Xia et al. identified BRPF1 as a potential drug target in lower-grade gliomas, as inhibiting BRPF1 function or silencing BRPF1 was found to reduce glioma cell proliferation and colony formation [60]. Lin et al. showed the USP35/BRPF1 axis promoted malignant features of PCa by activating the mevalonate pathway [61]. Although the oncogenic role of BRPF1 was clearly demonstrated in these studies, its relationship with drug resistance has not been questioned. Therefore, our study is the first to locate BRPFs in cancer drug resistance.

In order to phenocopy BRPF inhibition and rule out potential off-target activities, we depleted BRPF1 and BRPF2 by using RNAi. Based on the very few colonies growing after targeting BRPF2, we were able to test the taxane sensitivity only by targeting BRPF1 (Sup. Figure 10). Although it was not as potent as we have seen with inhibitors, downregulation of BRPF1 partially reversed taxane resistance (Fig. 4A, B). One of the explanations for this is that inhibitors may have a wider spectrum of action. While siRNA molecules are effective in decreasing the transcript level, they cannot affect protein levels that are already present in the cellular environment. In addition, the efficacy of multiple targets/signaling pathways that the drug has the potential to affect may not be achieved by targeting a single gene. Unfortunately, despite our several attempts, we failed to generate BRPF1 knockout-resistant cells as BRPF1 was very essential for the resistant phenotype. Another plausible explanation is drug engagement to ABCB1, inhibiting its efflux capacity. In the study of Barghout et al., it was demonstrated that various epigenetic probes, including the BRPF1 inhibitor PFI4, potentiate TAK-243 (a ubiquitin-activating enzyme inhibitor) cytotoxicity through off-target ABCG2 inhibition [62]. As anticipated, this potentiation did not result in significant alterations in the mode-of-action (i.e., ubiquitylation pathways) or expression levels of ABCG2. However, our study diverges in certain aspects and provides data contrary to the off-target modulation. First, the expression of ABCG2 was downregulated in Du145-CbzR (Log_2_FC: −2.03) cells according to our RNA-seq results, while it was not detected as a DEG in Du145-DtxR cells. Furthermore, CETSA analysis clearly showed that the BRPF inhibitors did not exhibit binding to the ABCB1 protein (Fig. 3H and Sup. Fig. 8E). Second, they were able to downregulate ABCB1 mRNA, indicating an interference at the transcriptional level (Fig. 3I). Third and most importantly, with the hint of transcriptional regulation, we screened several regions of the ABCB1 promoter and indeed showed that both endogenous and exogenous BRPF1 were enriched at the ABCB1 promoter (−658 bp) (Fig. 4D) suggesting direct regulation of ABCB1 via BRPF1. Therefore, the inhibition of BRPF, leading to a decrease in ABCB1 levels appears to play a crucial role in reversing taxane resistance in these cells, contrary to an off-target effect.

In order to evaluate the transcriptome changes induced by BRPF1 downregulation and understand how this may revert the resistance phenotype, an RNA-seq analysis was performed. We sought to find pathways that were differentially regulated in resistant cells and were reverted to more parental-like expression levels upon interference with BRPF1. Among the various pathways that fulfilled these criteria, we observed mTORC1 and UPR signaling pathways as the two most significant pathways showing alterations (Fig. 4G). We tested the essentiality of these pathways by using inhibitors. Both mTORC1 and UPR inhibition resensitized resistant cells to taxane and showed no activity on parental cells similar to BRPF inhibition (Fig. 4H, I and Sup. Fig. 12). mTOR signaling has been demonstrated to contribute to drug resistance, and the efficacy of its inhibitors has been investigated in clinical trials for PCa, including patients with advanced or resistant disease. Several studies, compiled by Avril et al., have demonstrated the significance of UPR in cancer chemotherapy resistance, while Bonsignore et al. highlighted its potential as a promising druggable target in cancer treatment [63, 64]. Further investigation is needed to test the contribution of the genes (listed in Sup. Tables 5 and 6) to drug resistance, considering their modulation by BRPF inhibition in these signaling pathways.

Through the examination of clinical data obtained from a pan-cancer study, we categorized patients into low and high BRPF1 expression groups, which revealed a significant association between elevated BRPF1 expression and reduced OS (Fig. 5A). While it was surprising to find lower BRPF1 expression in PCa among the analyzed cancer types, the elevated expression of BRPF1 in cases of local recurrence and metastasis suggests a potential contribution of BRPF1 to disease progression (Fig. 5B–D). Indeed, we observed a significant relationship between BRPF1 expression and PCa progression, as higher expression was associated with increased Gleason score and tumor stage, indicating its prognostic potential (Fig. 5E). Elevated BRPF1 expression was also observed in recurrent and metastatic cases (Fig. 5D), suggesting its role as a potential recurrence indicator. Patients with higher BRPF1 expression levels exhibit poor treatment response and more progressive disease (Fig. 5F), emphasizing its involvement in therapy outcome and disease progression. Intriguingly, an increase in BRPF1 expression was observed in patients exposed to taxane (Fig. 5F). Although we did not observe a significant upregulation of BRPF1 in our taxane-resistant cells (Sup. Fig. 14A), we hypothesize that parental cells have the potential to acutely upregulate BRPF expression in response to taxane exposure. Treatment with Dtx indeed led to a significant increase in BRPF1 expression in parental cells (Fig. 14B). In addition to the increase in BRPF1, we also observed an upregulation of ABCB1 expression following Dtx treatment. This suggests a potential interplay between BRPF1 and ABCB1, where the upregulation of BRPF1 may contribute to the subsequent upregulation of ABCB1, possibly through transcriptional regulatory mechanisms. In our study, we have provided evidence for the binding of BRPF1 to the ABCB1 promoter, supporting this proposed interaction. Further investigation is needed to elucidate the exact molecular signalings involved in this coordinated response.

## Conclusion

Our results showed that BRPF inhibition could serve as a promising strategy in taxane-resistant CR-PCa, and the mechanism of resensitization appears to involve the inhibition of drug efflux in cells that overexpress ABCB1. Since chemical inhibition of ABCB1 through small molecules is not feasible for cancer therapy due to high toxicity, revealing its regulators offers a good option for reducing its activity. In this context, our study herein is the first to identify BRPF1 as an ABCB1 regulator and uncover several epiregulators with the potential to reverse taxane resistance in CR-PCa.

### Supplementary information

Supplementary Figures

Supplementary Tables

Supplementary Figure and Table Legends

Supplementary Data-Uncropped WB Images

Supplementary Data-Original Data-qPCR

Reproducibility checklist

## Data Availability

The datasets generated during and/or analyzed during the current study are available in the GEO repository, under the accession number GSE247557.
