# Supplementary material for: Exploiting epigenetic targets to overcome taxane resistance in prostate cancer
Source: Cell Death Dis. 2024 Feb 12;15(2):132. doi: 10.1038/s41419-024-06422-1 (PMC10861560; doi:10.1038/s41419-024-06422-1)

**Sup. Fig. 1**


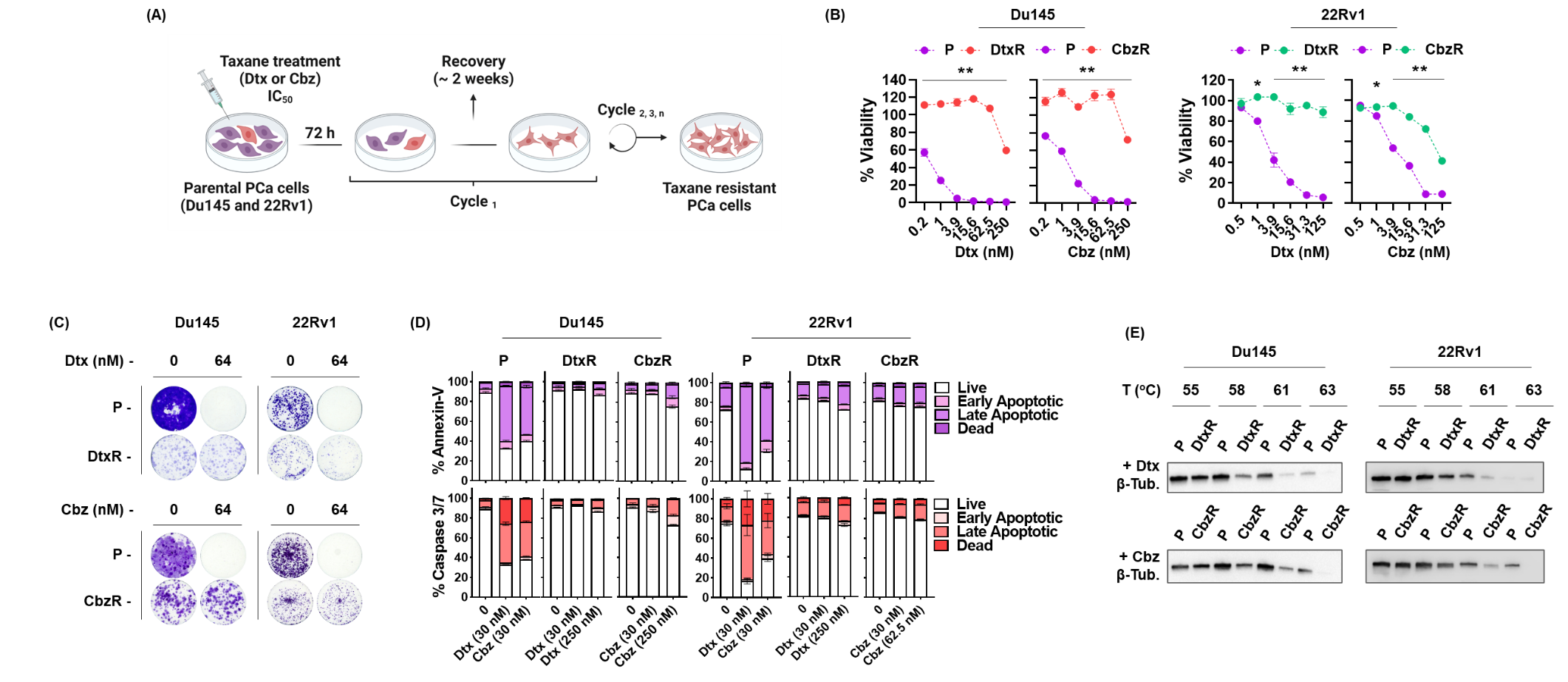


**Sup. Fig. 2**


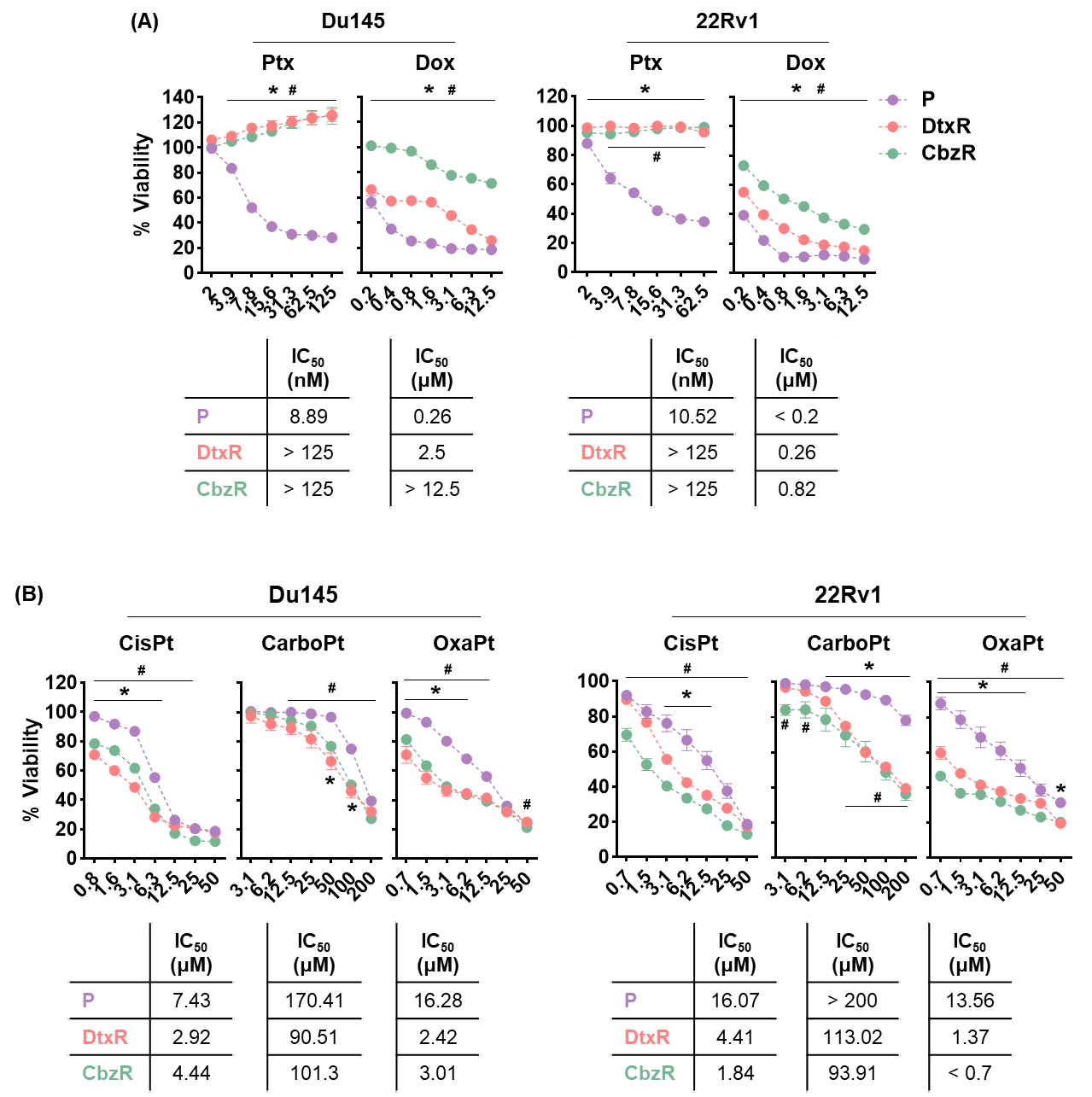


**Sup. Fig. 3**


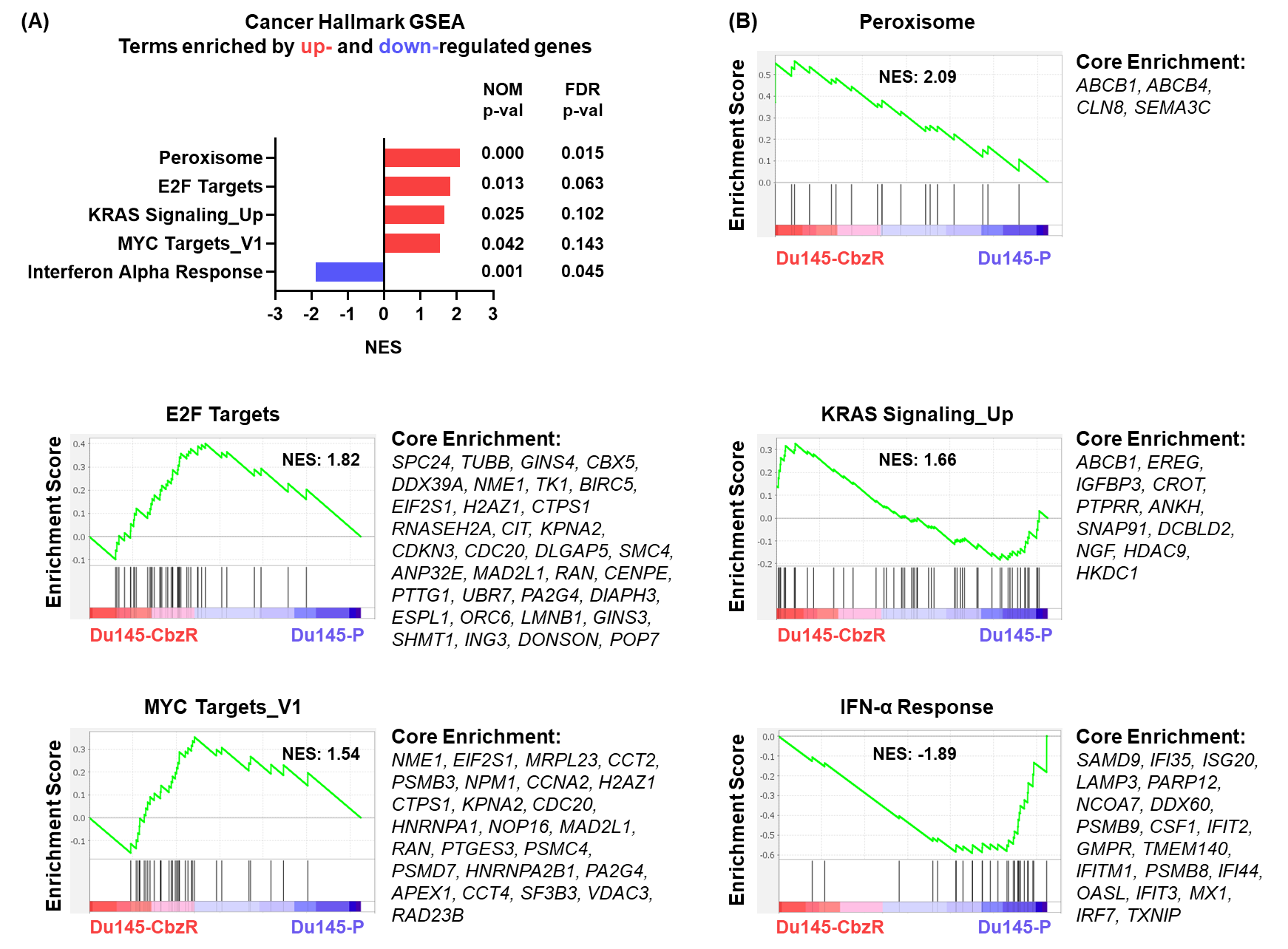


**Sup. Fig. 4**


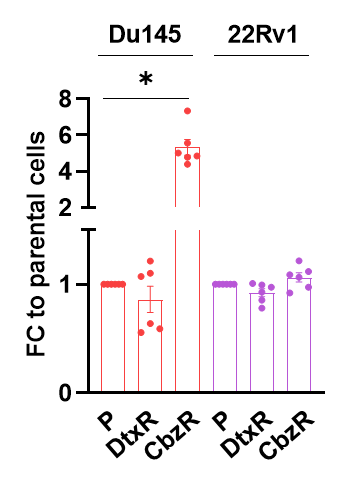


**Sup. Fig. 5**


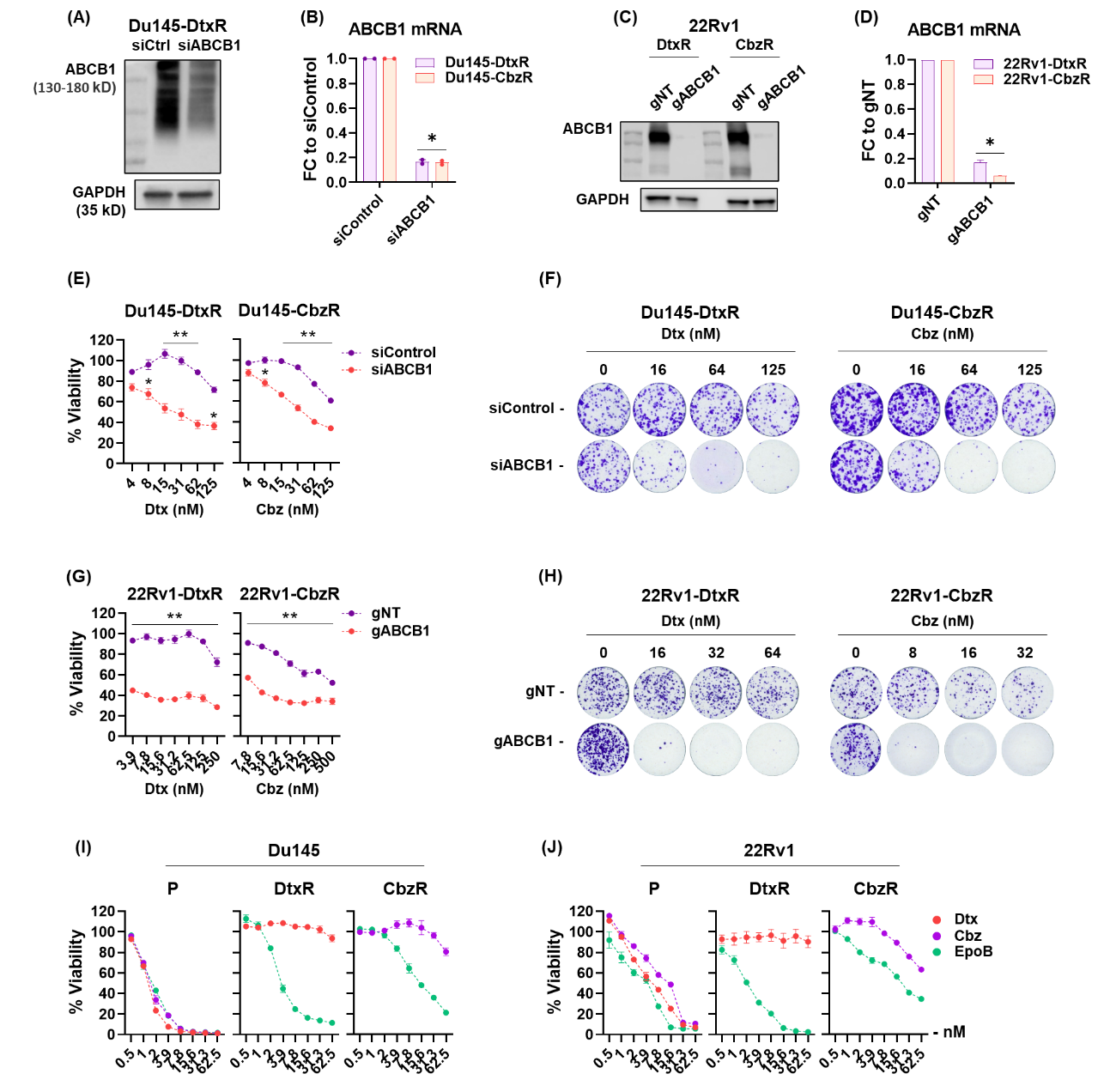


**Sup. Fig. 6**


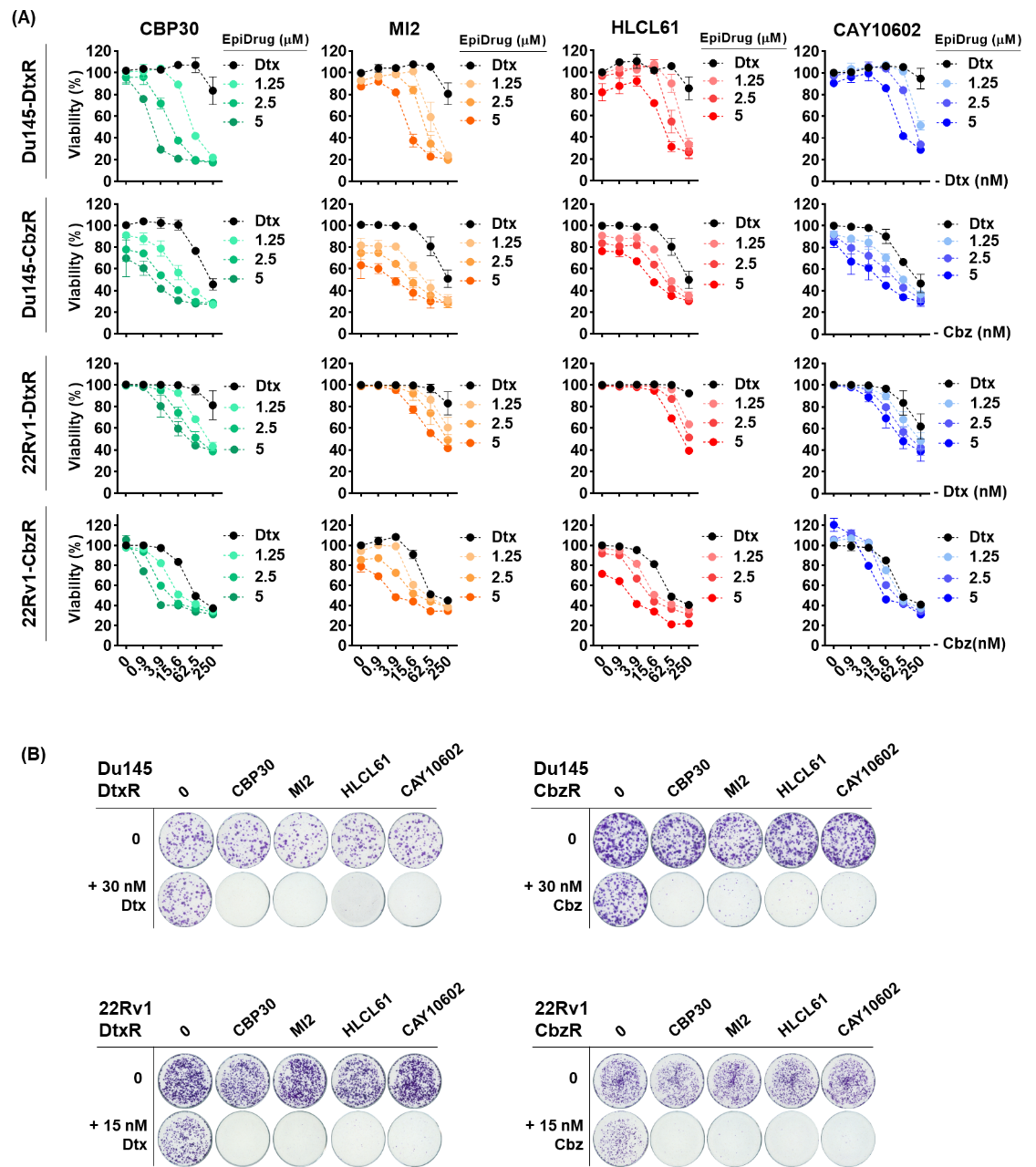


**Sup. Fig. 7**


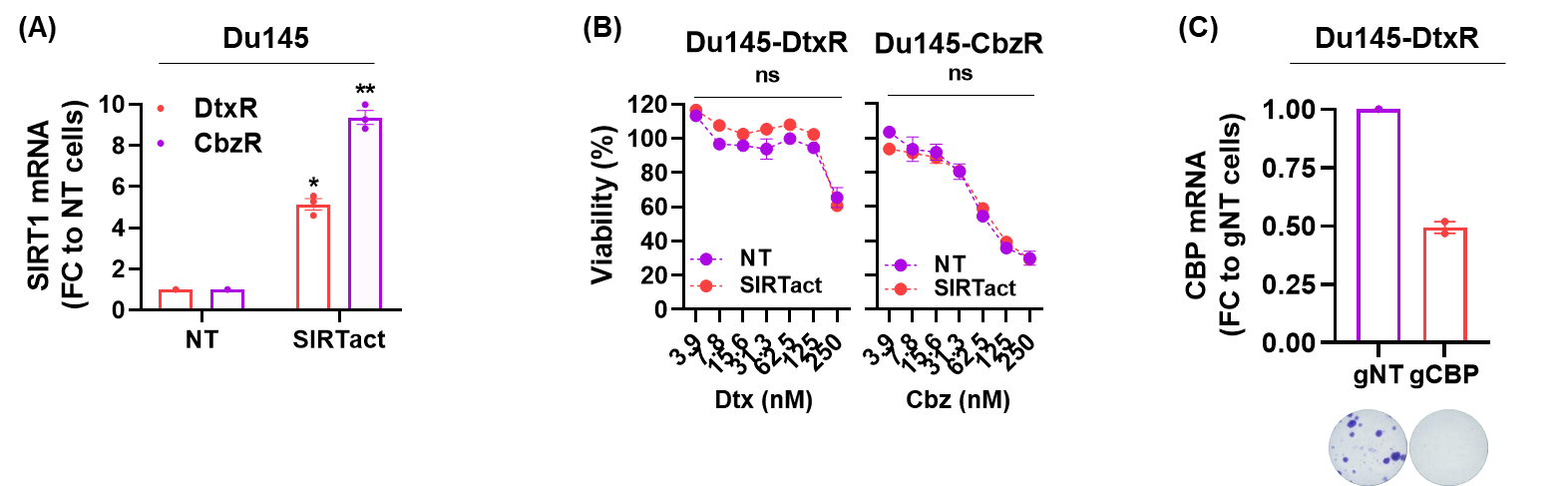


**Sup. Fig. 8**


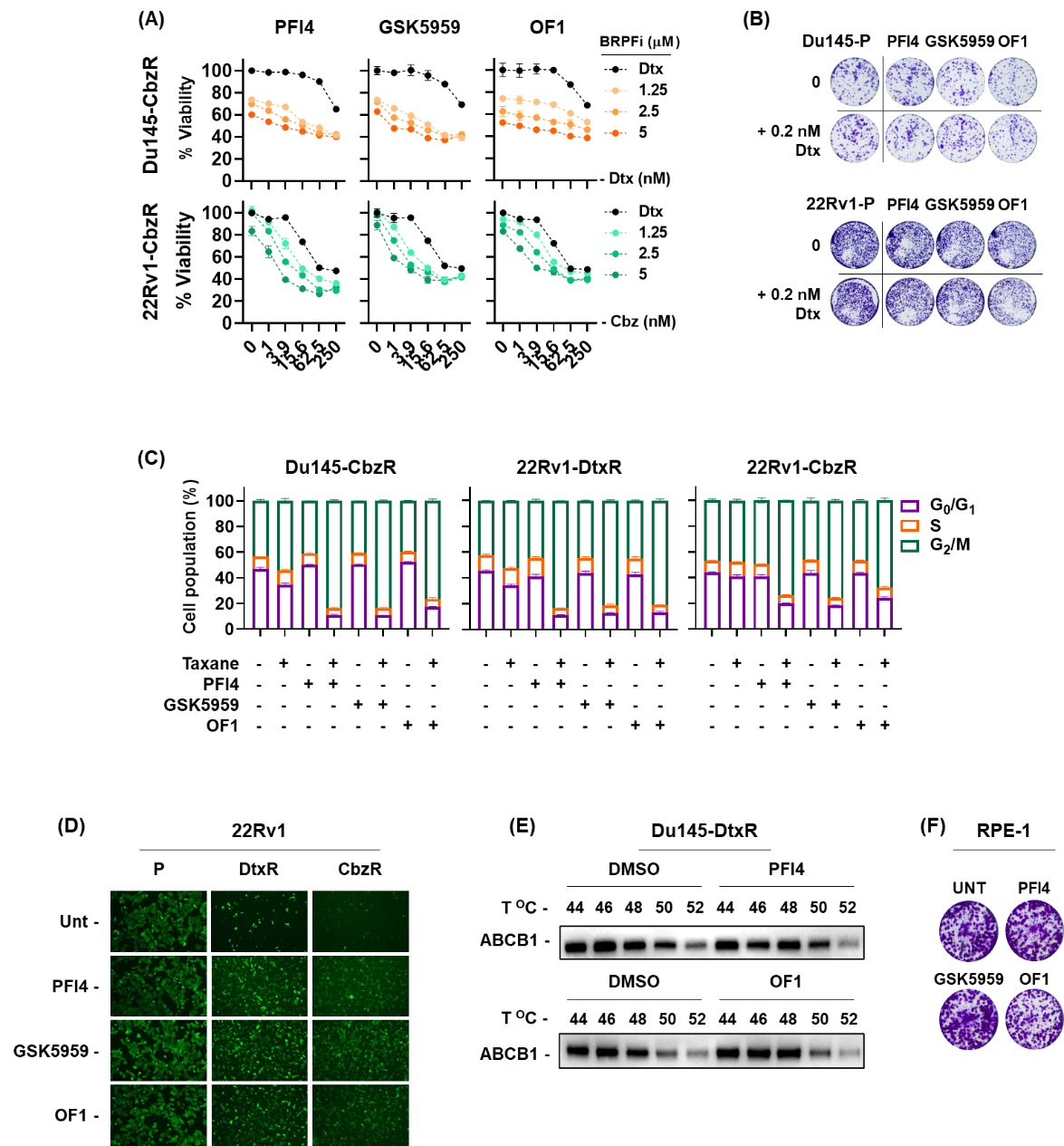


**Sup. Fig. 9**


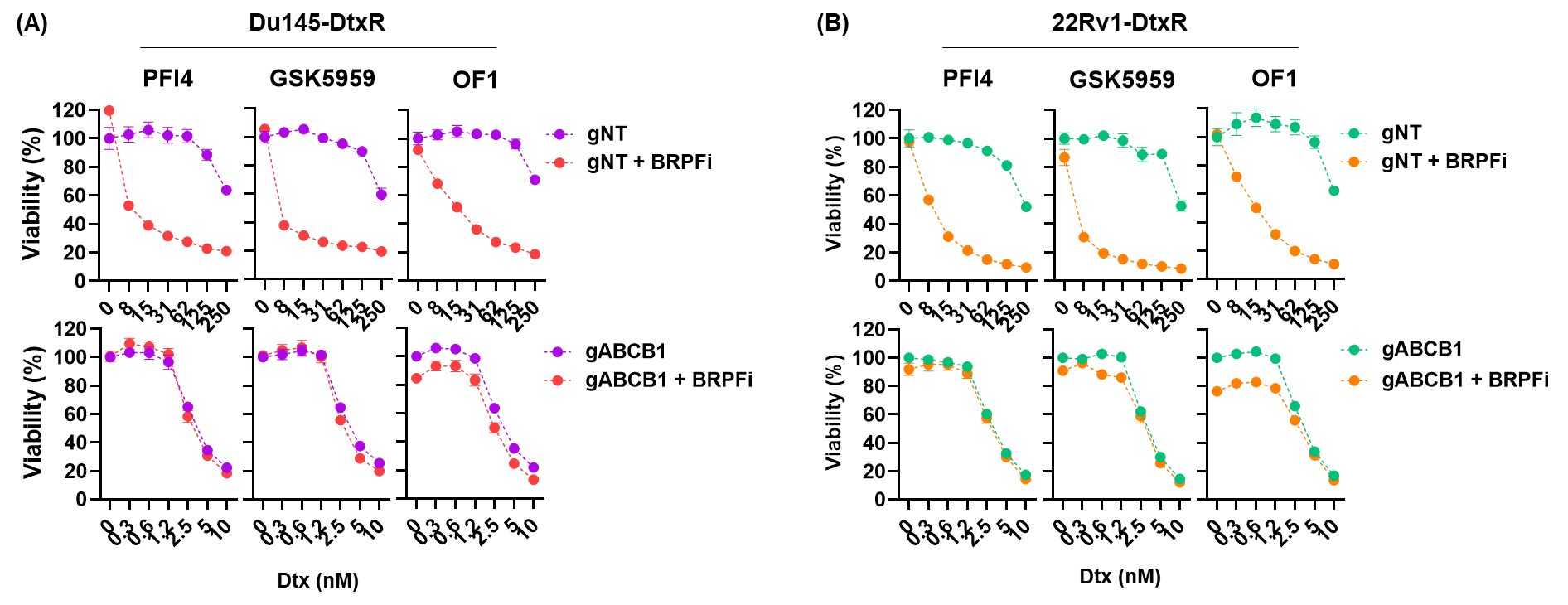


**Sup. Fig. 10**


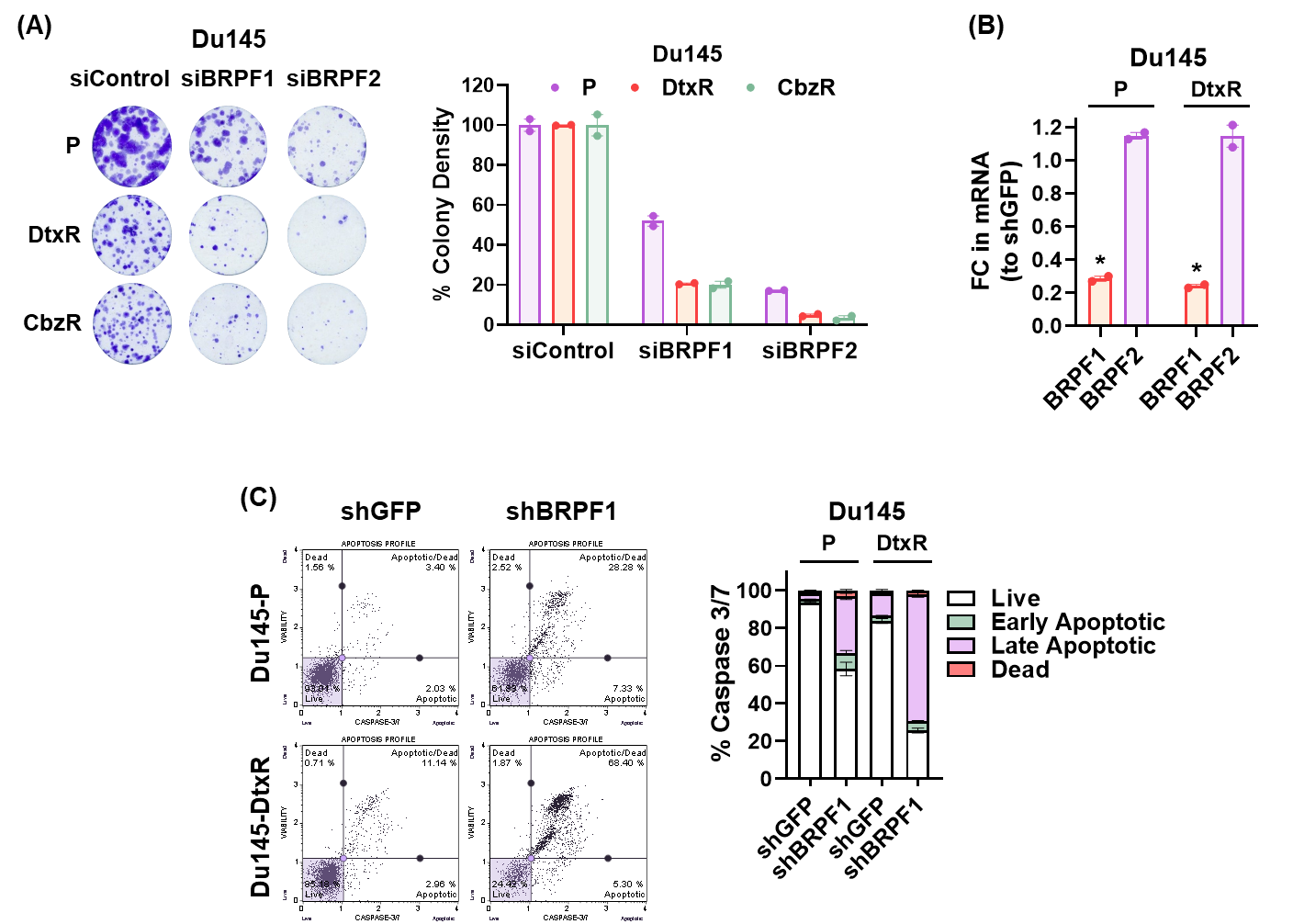


**Sup. Fig. 11**


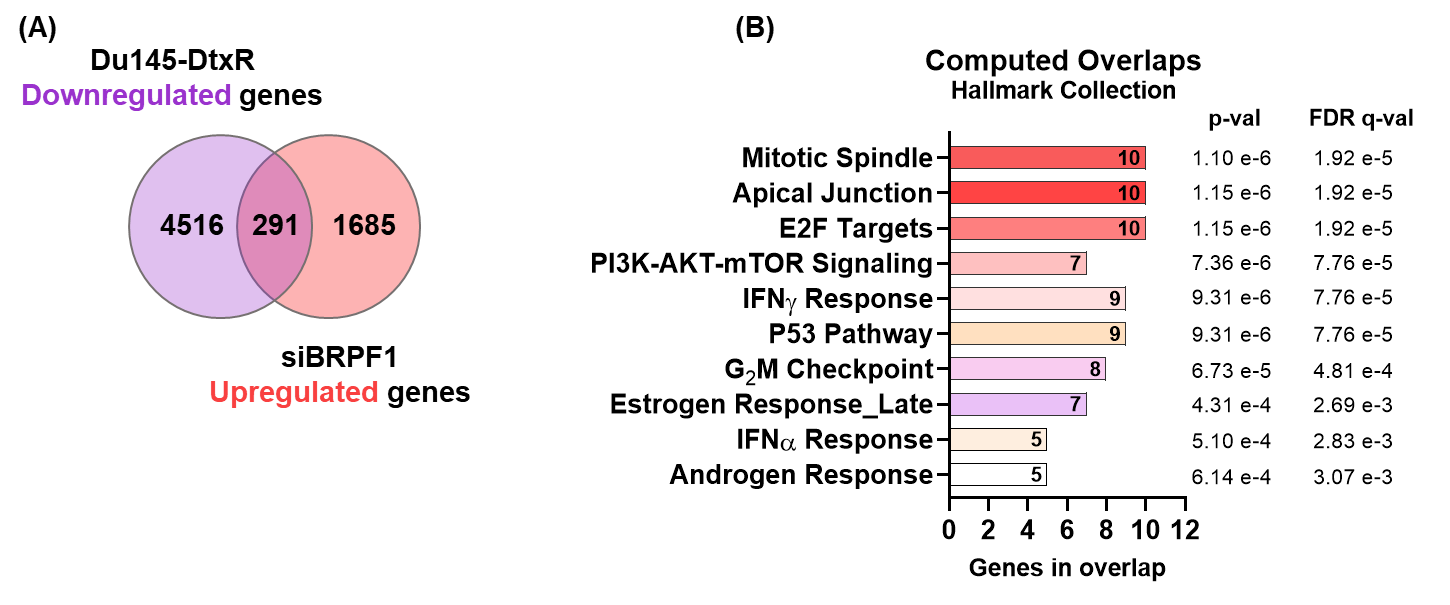


**Sup. Fig. 12**


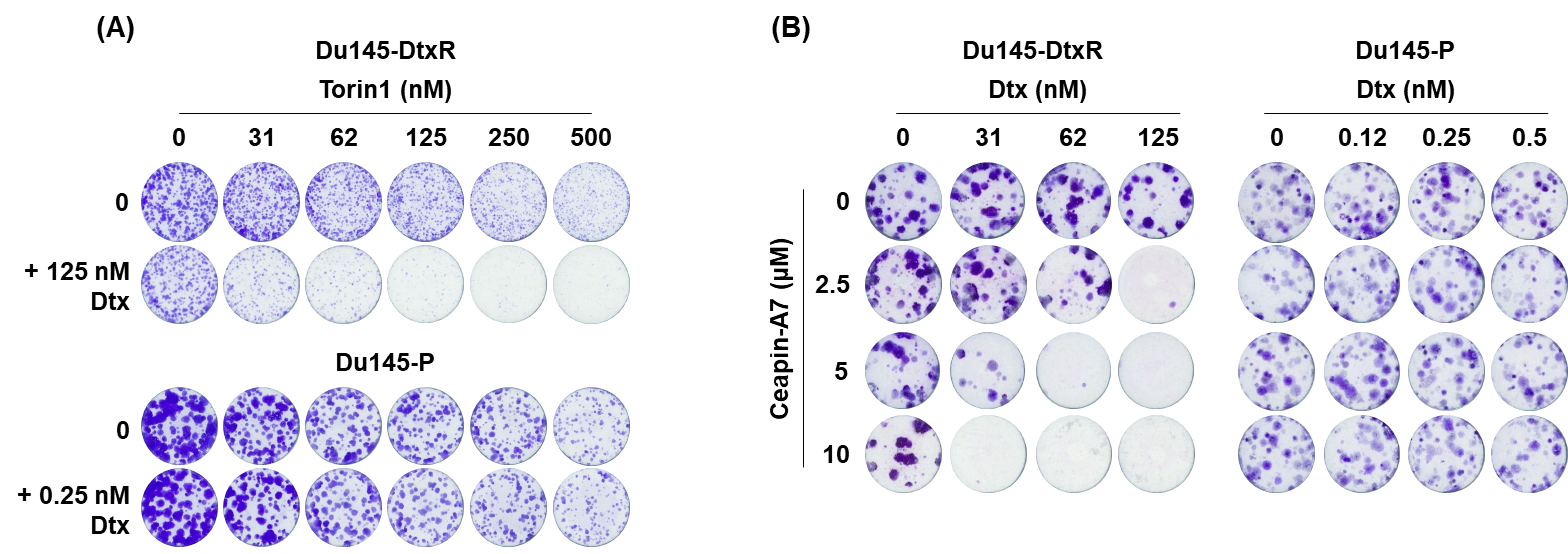


**Sup. Fig. 13**


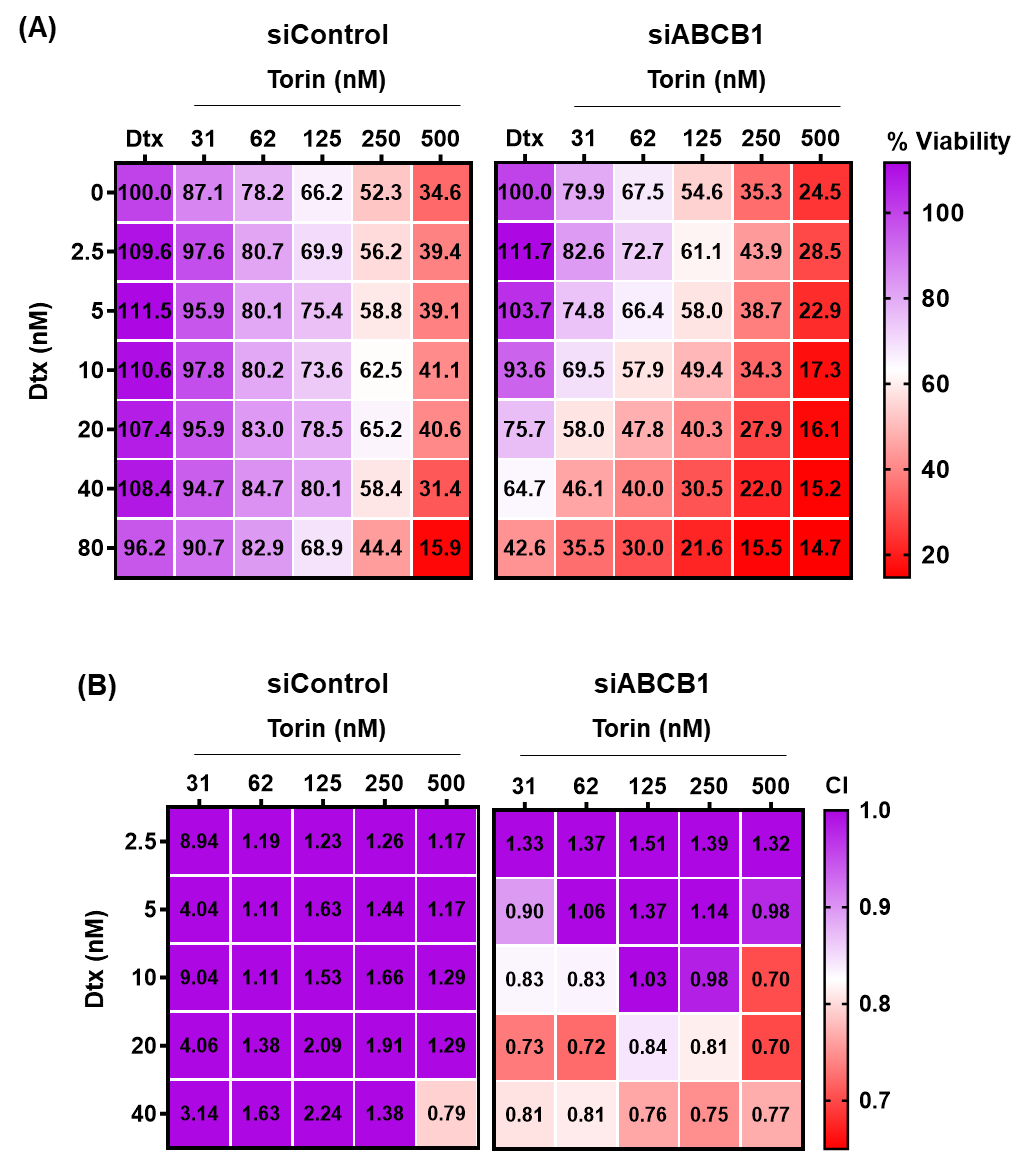


**Sup. Fig. 14**


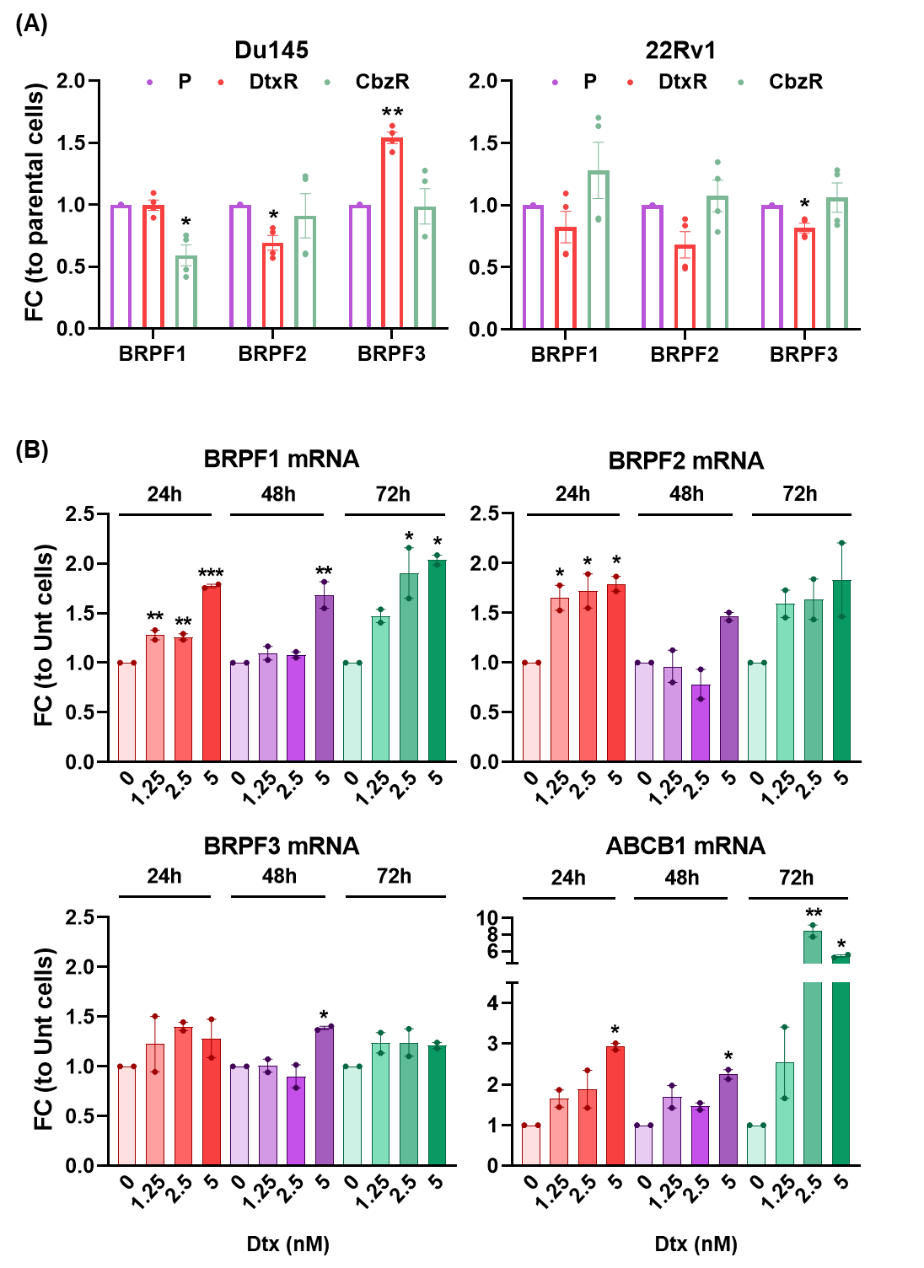

Supplement: Supplementary file 1 — Supplementary Figures [file 41419_2024_6422_MOESM1_ESM.docx]
