# Supplementary material for: Exploiting epigenetic targets to overcome taxane resistance in prostate cancer
Source: Cell Death Dis. 2024 Feb 12;15(2):132. doi: 10.1038/s41419-024-06422-1 (PMC10861560; doi:10.1038/s41419-024-06422-1)
Supplement: Supplementary file 2 — Supplementary Tables [file 41419_2024_6422_MOESM2_ESM.docx]

**Sup. Table 1.** The nucleotide sequences of the primers used in the study.

| **Gene Name** | **Forward Sequence (5’ to 3’)** | **Reverse Sequence (5’ to 3’)** |
| --- | --- | --- |
| **qRt-PCR** |  |  |
| **β-ACTIN** | TCACCATGGATGATGATATCGC | ATAGGAATCCTTCTGACCCATGC |
| **ABCA1** | ACAAGATGCTGAGGGCTGATG | CCAGTTTCTCCCTTGGTAGGG |
| **ABCA7** | GGTTCTATGGGCGGCTGAAG | TTGCATCCCACCAGAGAGGT |
| **ABCB1** | ACAGAGGGGATGGTCAGTGT | TCACGGCCATAGCGAATGTT |
| **ABCB4** | TCGCTGCTAAATCCAGGCAA | AACTCCAGCACCCAATCCTG |
| **BRPF1** | CGTGGAATTGATCCGCAAGC | GCTCCAAGGTTTTGCGAAGG |
| **BRPF2** | CAGCGGAAGAAGCAGTTTGTG | TCTTTGGCAGCCTTCATCTCC |
| **BRPF3** | AGAACATCGGCTATGACCCC | CACCTCTGGGGACAAATGGG |
| **CALR** | CGCTTTTATGCTCTGTCGGC | CCACAGATGTCGGGACCAAA |
| **CCN1** | CGCCTTGTGAAAGAAACCCG | GGTTCGGGGGATTTCTTGGT |
| **CCN2** | GGTGTGGCTTTAGGAGCAGT | TGATGGCTGGAGAATGCACA |
| **CLN8** | ATGTGCTCACGCAGACAGAA | AGGACAGAGCACAAAGGAGC |
| **CREBBP** | CAGTTTGCCCACCTTCCCTA | GCTGCTGTATCAGTTTGCGT |
| **CROT** | AGTCCAATTCCCGCACCTTT | ACTAAGTCGGTGACTGTCCG |
| **CXCL8** | AACTTTCAGAGACAGCAGAGCA | TGGTTCCTTCCGGTGGTTTC |
| **DCBLD2** | TGACTTGTAATGAGCAGGGGG | ATGGGGCAAGACACCAAAGG |
| **DDX39A** | CGGCGGAAAACCGAAGTTG | AGCTGTGGATGGAAACGTAGG |
| **DIAPH3** | CATTCAGTGGTCCTGTGCCT | ACCCAAATGGCAGGATTGGT |
| **EDN1** | AGGAGTTCCCCCAACCATCT | GAGGTCCATTGTCATCCCCA |
| **EGR1** | TCCCATTTACTCAGCGGCAC | TGGAAACAGGTAGTCGGGGA |
| **EP300** | TCCCCCTCAAAAATGCTGGT | AACAGTGCTTAGGTTGGGGG |
| **EREG** | CCAGGTCAGGGAGGATCTGT | CGTGTGATTGCACCAGGGTA |
| **EIF2S1** | ACTACGACAACCCTGGAGAGA | TCTGCCTCGCAAGTTCAGTC |
| **FOSL1** | CTCTGGAGCCCTAACCCCTT | GTTGGGTGGATCACAGGAAGA |
| **GADD45A** | CGGCCCAATTAGTGTCGTG | TTTCTGCACTCACTCACAGGCfx |
| **GRWD** | ACTCCCACCCAGAGACTTGT | GTTCTGGGGACACAGGTCAA |
| **HERPUD1** | GGAGGCTTTGACAGGAATGGA | ACGGCTTCACGTTTCTGCT |
| **HSPA1A** | TGCGACAGTCCACTACCTTTT | GCTGGAAACGGAACACTGGAT |
| **HSPA5** | GGGTCATGTGCATCTGGTGT | ACCAAATACTCCCTCCCCTCA |
| **HSPA8** | TGGCACCACCTACTCTTGTG | TTGGAGTGGTTCGGTTTCCC |
| **HSP90AA1** | ACATCTGCCTCTGGTGATGAG | CCTGGTCCTTGGTCTCACCT |
| **IRF7** | CGGCTGGAAAACCAACTTCC | CTGAGCGCGTACACCTTGT |
| **JUN** | TAATCCAGTCCAGCAACGGG | GTGTTCTGGCTGTGCAGTTC |
| **JPT1** | CAGCAGGTGCCAAGTCTAGT | GGAGCTTGCTTCAGAGGAGTT |
| **MAD2L1** | AATCGTGGCCGAGTTCTTCT | TTACAAGCAAGGTGAGTCCGT |
| **MARCKS** | ACCATTCCAACAGGTCGAGG | TCCCTTTTTGACCCACCCAT |
| **MRPL23** | GGTGCAGCATGGCTCTAACA | CGTAGGCGACCTTGTAGTCC |
| **MYC** | TTCCCCTACCCTCTCAACGA | CAGAGAATCCGAGGACGGAG |
| **MX1** | CAGACTCCGACACGAGTTCC | AAAGCCTGGCAGCTCTCTAC |
| **NFKB1** | TAATGCCTTCCGGCTGAGTC | TGCTTCGGTGTAGCCCATTT |
| **ODC1** | GGCACACGCAGAGGGATTT | GTCCAGAATGTCCTTGGCAGTA |
| **PLK2** | AGGTGTTGACAGAGCCAGAAG | CAGACCGAAGTCCCCAACTTT |
| **PRMT3** | TGTCAGAACCTGCTCGTCAT | TCTCGGTAGCTTTCTGTTCGT |
| **PTPRR** | TGTTGTCGTGGACCCTCAAG | AAGGGCAAACAGAGGTAGCG |
| **RAN** | ACGACTTAGAGGTTGCTCAGAC | TAGACTTCTGACGCTGGGCT |
| **SERP1** | TCAGCCTAAAGTGGCATCTGG | AAACCTGCCCTATGACCCTC |
| **SEMA3C** | ATGGCTGGCAAAGATCCCAC | GCCACTCCCACAGACATACAA |
| **SIRT1** | ACATAGACACGCTGGAACAGG | TCCTCGTACAGCTTCACAGTC |
| **SLC4A4** | AGCCAACAAGTCCAAACCGA | CCAACAAAGCCGGGATAGCA |
| **SPC24** | AGGAGGACACCCGTCTGAA | GATCCGCCTCAATCTCCTTGA |
| **SRM** | GTCTGTCTCTCTGGCGTTCC | GTGAGTGAGGGGCAACAGAA |
| **STC2** | GCCATTGTACTGTCTGGGCT | ACCCCCTTTGAGCTGGTTTT |
| **TNFAIP3** | CAGAACACCATTCCGTGCCT | GTGCTTTGTGTGGTTCGAGG |
| **TXNIP** | AGCCAGCCAACTCAAGAGAC | CCGCCCATCAGGAATGAACA |
| **qRt-PCR from Genomic DNA** | |  |
| **ABCB1 gDNA** | AGATCTACCAGGACGAGTGAGAAAA | AACAGTCAGTTCCTATATCCTGTGTCT |
| **GAPDH gDNA** | AAGAAGATGCGGCTGACTGT | CGGCTACTAGCGGTTTTACG |
| **ChIP-qPCR** | |  |
| **Chr 12 Gene Desert** | TGTACAGGGCCTGGTTACCCACA | AGGAGGGCCTAGCTGGTGTCAT |
| **RUNX2** | GGAAACGGAGGGGTCTGAAC | TAAGAGCTGGTTTCGCCGTC |
| **ABCB1-primer pair 1 (-130)** | CCCTAAGCCATGTAACTCTTCG | TGACTATGGCTCCAAAGCAT |
| **ABCB1-primer pair 2 (-234)** | GGTCTTCCCAGTAACCTACCA | ACATGGCTTAGGGATTGGGG |
| **ABCB1-primer pair 3 (-658)** | TTCTCTCTGTGACAGCTCAGT | AGCACAAATTGAAGGAAGGAGT |
| **CRISPR-Cas9**  **Non-targeting gRNA**  **ABCB1 gRNA**  **CREBBP gRNA** | GTATTACTGATATTGGTGGG  TTGGACTGTCAGCTGCTGTC  CACCGAGCGGCTCTAGTATCAACCC | CCCACCAATATCAGTAATAC  GACAGCAGCTGACAGTCCAA  AAACGGGTTGATACTAGAGCCGCTC |

**Sup. Table 2.** Top 10 ranked up- (red) and down-regulated (purple) genes in Du145-DtxR cells.

| Gene Name | LogFC | p-val | FDR | Description |
| --- | --- | --- | --- | --- |
| CP | 10.7 | 9.5E-107 | 2.5E-104 | Ceruloplasmin |
| ABCB1 | 9.1 | 3.7E-91 | 7.9E-89 | ATP Binding Cassette Subfamily B Member 1 |
| ABCC9 | 8.9 | 1.7E-12 | 1.7E-11 | ATP Binding Cassette Subfamily C Member 9 |
| KRTAP2-3 | 8.3 | 3.4E-96 | 7.6E-94 | Keratin Associated Protein 2-3 |
| C4orf19 | 7.6 | 4.6E-10 | 3.7E-09 | Chromosome 4 Open Reading Frame 19 |
| CFAP299 | 6.3 | 6.4E-07 | 3.5E-06 | Cilia And Flagella Associated Protein 299 |
| TDRD1 | 6.0 | 1.7E-06 | 9.2E-06 | Tudor Domain Containing 1 |
| GJA1 | 5.9 | 7.7E-101 | 1.8E-98 | Gap Junction Protein Alpha 1 |
| MIR4500HG | 5.7 | 1.7E-06 | 9.1E-06 | MIR4500 Host Gene |
| NFE4 | 5.7 | 1.9E-14 | 2.3E-13 | Nuclear Factor, Erythroid 4 |
| NUDT7 | -8.3 | 2.1E-11 | 1.9E-10 | Nudix hydrolase 7 |
| CA12 | -8.4 | 1.6E-11 | 1.5E-10 | Carbonic anhydrase 12 |
| TSNARE1 | -8.6 | 7.6E-12 | 7.3E-11 | t-SNARE domain containing 1 |
| SLC6A11 | -8.9 | 1.0E-12 | 1.0E-11 | Solute carrier family 6 member 11 |
| ADAMTS15 | -9.0 | 6.0E-13 | 6.3E-12 | ADAM metallopeptidase with thrombospondin type 1 motif 15 |
| COL5A1 | -9.0 | 4.8E-13 | 5.1E-12 | Collagen type V alpha 1 chain |
| S1PR3 | -9.5 | 2.6E-14 | 3.0E-13 | Sphingosine-1-phosphate receptor 3 |
| DAPK1 | -10.3 | 2.4E-16 | 3.3E-15 | Death associated protein kinase 1 |
| SH3BGRL | -11.2 | 6.0E-19 | 9.8E-18 | SH3 domain binding glutamate rich protein like |
| BCHE | -12.3 | 4.3E-22 | 8.8E-21 | Butyrylcholinesterase |

**Sup. Table 3.** Top 10 ranked up- (red) and down-regulated (purple) genes in Du145-CbzR cells.

| Gene Name | LogFC | p-val | FDR | Description |
| --- | --- | --- | --- | --- |
| ABCB1 | 12.0 | 1.1E-154 | 1.7E-151 | ATP Binding Cassette Subfamily B Member 1 |
| SLFN14 | 6.9 | 1.E-23 | 8.5E-22 | Schlafen Family Member 14 |
| HNRNPA1P9 | 6.7 | 2.4E-07 | 2.2E-06 | Heterogeneous Nuclear Ribonucleoprotein A1 Pseudogene 9 |
| CCDC187 | 6.0 | 2.0E-25 | 1.0E-23 | Coiled-Coil Domain Containing 187 |
| ABCB4 | 5.9 | 4.4E-15 | 1.0E-13 | ATP Binding Cassette Subfamily B Member 4 |
| MMP1 | 5.8 | 2.8E-89 | 1.2E-86 | Matrix Metallopeptidase 1 |
| ENSG00000286432 | 5.7 | 1.8E-23 | 8.1E-22 | lncRNA, novel transcript |
| MIR4500HG | 5.7 | 3.3E-06 | 2.5E-05 | MIR4500 Host Gene |
| MC5R | 5.4 | 8.8E-06 | 6.3E-05 | Melanocortin 5 Receptor |
| LINC01322 | 5.3 | 1.7E-43 | 2.2E-41 | Long Intergenic Non-Protein Coding RNA 1322 |
| EVC2 | -8.3 | 2.1E-11 | 1.9E-10 | EvC Ciliary Complex Subunit 2 |
| GPR173 | -8.3 | 1.6E-11 | 1.5E-10 | G Protein-Coupled Receptor 173 |
| DNAJC15 | -8.5 | 7.6E-12 | 7.3E-11 | DnaJ Heat Shock Protein Family (Hsp40) Member C15 |
| ZNF677 | -8.6 | 1.0E-12 | 1.0E-11 | Zinc Finger Protein 677 |
| TXNIP | -8.6 | 6.0E-13 | 6.3E-12 | Thioredoxin Interacting Protein |
| BRDT | -8.6 | 4.8E-13 | 5.1E-12 | Bromodomain Testis Associated |
| TINAGL1 | -10.3 | 2.6E-14 | 3.0E-13 | Tubulointerstitial Nephritis Antigen Like 1 |
| SLFN11 | -10.4 | 2.4E-16 | 3.3E-15 | Schlafen Family Member 11 |
| ZNF518B | -10.5 | 6.0E-19 | 9.8E-18 | Zinc Finger Protein 518B |
| BCHE | -12.8 | 4.3E-22 | 8.8E-21 | Butyrylcholinesterase |

**Sup. Table 4.** The genes in positively (NES ≥ 1.5) and negatively (NES ≤ 1.5) enriched gene sets in Du145-DtxR cells.

| **Gene Sets** | **Genes** |
| --- | --- |
| **MYC Targets v1** | *ODC1, CANX, ABCE1, EIF2S1, SYNCRIP, NOP16, TCP1, NOLC1, DDX18, IARS1, H2AZ1, SRM, SRPK1, MRPS18B, RAN, MAD2L1, PSMD14, HSPE1, SNRPA1, IFRD1, COPS5, HNRNPA3, CTPS1, SF3B3, ETF1, EPRS1, CAD, KARS1, MRPL23, HDGF, CCNA2, DDX21, CCT2, CCT3, NPM1, GOT2, HNRNPD, PSMD1, EIF4E, XPOT, CCT5, BUB3, NOP56, CCT4, HSPD1, EIF1AX, HSP90AB1, EIF3J, ORC2, PSMB2, C1QBP, XRCC6, PSMD7, EIF4A1, AP3S1, VBP1, KPNA2, EIF4G2, NME1, SSB, CDC20, CSTF2, CCT7, SERBP1, PSMA6, VDAC3, PA2G4, SNRPG, PSMA1, PRDX4* |
| **UPR** | *HERPUD1, HSPA5, STC2, DNAJC3, CALR, HYOU1, HSP90B1, XBP1, SERP1, CHAC1, EIF2S1, DNAJB9, EIF4EBP1, TUBB2A, SSR1, DKC1, NOP14, PDIA6, CEBPG, NOLC1, VEGFA, IARS1, SLC7A5, DDX10, TARS1, SPCS3, SEC31A, LSM1, ATF3, PSAT1, TATDN2, ATP6V0D1, NABP1, ASNS, SRPRB, DDIT4, ERN1, NPM1, EIF4E, XPOT, NOP56, HSPA9, GEMIN4, MTHFD2* |
| **MYC Targets v2** | *HK2, TFB2M, NDUFAF4, NIP7, NOP16, MRTO4, NOLC1, GRWD1, DDX18, SRM, RRP12, PPRC1, BYSL, UTP20, HSPE1, FARSA, WDR43, DCTPP1, WDR74, NPM1, IMP4, PES1, PUS1, PLK4, MYBBP1A, NOP56, HSPD1, PLK1, NOC4L, SUPV3L1, PRMT3* |
| **E2F Targets** | *LYAR, JPT1, EIF2S1, SYNCRIP, EXOSC8, DDX39A, TUBB, NOLC1, DIAPH3, H2AZ1, PSMC3IP, HMGA1, MELK, RAN, MAD2L1, SMC4, PRIM2, TACC3, CTPS1, GINS4, TIPIN, DCTPP1, HNRNPD, ORC6, ING3, PLK4, NOP56, ASF1A, ORC2, HUS1, DONSON, MTHFD2, XRCC6, PLK1, PNN, ANP32E, ZW10, CDKN3, CDC25A, KPNA2, PRKDC, CSE1L, NME1, TRIP13, CENPE, CDC20, CDKN1A, NUDT21, CKS1B, RAD51AP1, EZH2, PA2G4, PRDX4* |
| **TNFA Signaling via NFKB** | *BCL2A1, EDN1, ICAM1, SERPINB8, FOSL1, TNC, TNFAIP3, JUN, NAMPT, CXCL1, G0S2, CXCL2, GADD45A, IL15RA, IL7R, MXD1, CD44, PLK2, EGR1, HBEGF, CSF2, ETS2, KLF6, MSC, ATP2B1, CFLAR, TNFRSF9, IL6, DUSP5, MAP2K3, CCND1, DNAJB4, GADD45B, TUBB2A, MARCKS, CCN1, SGK1, CSF1, SERPINE1, CCL20, TLR2* |
| **Inflammatory Response** | *EDN1, SLC4A4, CXCL8, ICAM1, CYBB, NAMPT, DCBLD2, IL18R1, IL15RA, RNF144B, EREG, IL7R, MXD1, P2RY2, CD70, HBEGF, HRH1, KLF6, ATP2B1, TNFRSF9, IL6, LPAR1, AXL, SLC7A2, TIMP1, LYN, CSF1, SERPINE1, CCL20, TLR2* |
| **IL6-JAK-STAT3 Signaling** | *FAS, JUN, CXCL1, IL18R1, IL15RA, CD44, CSF2, IL6, IL7, TNFRSF12A* |
| **MTORC1 Signaling** | *DDIT3, HSPA5, SDF2L1, BCAT1, NAMPT, CALR, HSP90B1, NIBAN1, ARPC5L, CANX, MLLT11, PDK1, XBP1, SERP1, STARD4, FKBP2, HK2, POLR3G, CTSC, MTHFD2L, MAP2K3, GMPS, PSMG1, WARS1, SSR1, CACYBP, TRIB3, PPA1, EEF1E1, ATP6V1D, UCHL5, DDX39A, PNO1, ITGB2, SLC7A5, PSME3, GOT1, DHCR24, SLC7A11, PSMD12, PSAT1, PSMD14, TCEA1, HSPE1, IFRD1, COPS5, NFKBIB, RIT1, ASNS, SLC2A1, ETF1, PSMA3, EPRS1, ACTR3, SLC1A5, DDIT4, ELOVL6, SHMT2* |
| **G_2_M Checkpoint** | *SAP30, ODC1, HSPA8, JPT1, SYNCRIP, CCND1, DKC1, MARCKS, DDX39A, KMT5A, FBXO5, NOLC1, SLC7A5, H2AZ1, HMGA1, E2F3, E2F4, MAD2L1, SMC4, PRIM2, TACC3, ORC5, ATF5, CCNA2, CDC6, HNRNPD, ORC6, PLK4, SLC7A1, BUB3, NOTCH2, DR1, HUS1, KIF20B, PLK1, CDKN3, CDC25A, RBM14, KPNA2, NUP50, CENPE, CDC20, HIF1A, NSD2* |
| **Allograft Rejection** | *ICAM1, IL12A, FAS, BCAT1, EREG, CAPG, ETS1, PSMB10, IL6, IL7, TAP2, ABCE1, WARS1, TIMP1, LYN, CSF1, TLR2, HDAC9, ITGB2, BCL10, EIF5A* |
| **IL2-STAT5 Signaling** | *ABCB1, IL1RL1, SCN9A, PLAGL1, IL18R1, ODC1, CAPG, MXD1, CD44, CSF2, SPRY4, NT5E, KLF6, NFKBIZ, CDCP1, TNFRSF9, PRKCH, XBP1, ITGA6, BCL2L1, PDCD2L, HK2* |
| **Estrogen Response_Late** | *IMPA2, SCNN1A, ALDH3B1, IDH2, TFPI2, OPN3, CCN5, FGFR3, SEMA3B, CYP26B1, CISH, CELSR2, TOB1, TJP3, FOS, ASS1, DNAJC12, TNNC1, TFAP2C, CACNA2D2, RET, IGFBP4, CDH1, OLFM1, CYP4F11, CA12* |
| **KRAS Signaling_Dn** | *ABCG4, ATP6V1B1, IGFBP2, GAMT, PDK2, SLC30A3, RYR1, PDE6B, ADRA2C, TFCP2L1, YBX2, HTR1B, FGFR3, CD207, CACNA1F, ARHGDIG, PTPRJ, CELSR2, PRODH, EGF, YPEL1, LYPD3, NR4A2, TNNI3, KCNN1, RGS11, EFHD1, LFNG, MX1, P2RX6, SNCB, KCNQ2, SLC38A3, SELENOP, GPRC5C* |
| **Angiogenesis** | *POSTN, FSTL1, S100A4, MSX1, VTN, SPP1, THBD, COL3A1, STC1* |

**Sup. Table 5.** Representation of overlapping pathways/signaling of genes downregulated by siBRPF1 (The set of genes which lie in the intersection of the Venn diagram shown in **Figure 4F.**

| **Gene Set Name** | **Description** | **Genes** |
| --- | --- | --- |
| **Unfolded Protein Response** | Genes upregulated during unfolded protein response; a cellular stress response related to the endoplasmic reticulum | *HSPA5, DDIT4, XBP1, ASNS, MTHFD2, HSP90B1, CALR, SERP1, PSAT1, EDEM1, ATF3, NOLC1, CKS1B, SHC1, SRPRB, HYOU1, EIF4EBP1, ERN1, ATF4, ALDH18A1, CHAC1, HERPUD1* |
| **MTORC1 Signaling** | Genes upregulated through activation of mTORC1 complex | *HSPA5, DDIT4, XBP1, ASNS, MTHFD2, HSP90B1, CALR, SERP1, PSAT1, EDEM1, LRX, SLC2A1, DDIT3, SLC1A5, ITGB2, TRIB3, STARD4, CYB5B, IDH1, GOT1, WARS1, TCEA1, POLR3G, SDF2L1, UFM1* |
| **TNFA Signaling via NF-κB** | Genes regulated by NF-kB in response to TNF | *ATF3, PLAUR, SERPINE1, CCN1, CXCL1, MYC, TNFRSF9, HBEGF, GCH1, ICAM1, RIPK2, IL18, IL15RA, RELA, FOSL2, TRAF1, PHLDA1, PLAU, BIRC3, DUSP5, EGR1, BIRC2, FOSL1* |
| **Epithelial Mesenchymal Transition** | Genes defining epithelial-mesenchymal transition, as in wound healing, fibrosis, and metastasis | *PLAUR, SERPINE1, CCN1, CXCL1, CXCL8, PVR, CCN2, LOX, ITGA2, COL4A2, GJA1, THBS1, DST, DKK1, LAMC2, CTHRC1, COL12A1, LRRC15, LOXL2, TPM4, VEGFC, IL32* |
| **Inflammatory Response** | Genes defining inflammatory response | *PLAUR, SERPINE1, MYC, TNFRSF9, HBEGF, GCH1, ICAM1, RIPK2, IL18, IL15RA, RELA, CXCL8, PVR, SLC7A2, SLC7A1, LAMP3, IRAK2, CD70* |
| **Hypoxia** | Genes upregulated in response to low oxygen levels (hypoxia) | *HSPA5, DDIT4, ATF3, GLRX, SLC2A1, DDIT3, PLAUR, SERPINE1, CCN1, FOSL2, CCN2, LOX, ERRFI1, CP, PAM, HOXB9* |
| **IL2 STAT5 Signaling** | Genes upregulated by STAT5 in response to IL2 stimulation | *XBP1, SLC1A5, MYC, TNFRSF9, TRAF1, PHLDA1, DHRS3, FAM126B, PLAGL1, PRKCH, SLC39A8, CDCP1, SPRY4, SH3BGRL2* |
| **KRAS Signaling_Up** | Genes upregulated by KRAS activation | *GLRX, ITGB2, PLAUR, HBEGF, TRAF1, PLAU, BIRC3, ITGA2, DUSP6, HDAC9, ETV5, DNMBP, TMEM158, GALNT3* |
| **Cholesterol Homeostasis** | Genes involved in cholesterol homeostasis | *ATF3, TRIB3, STARD4, PLAUR, ERRFI1, MAL2, GLDC, GPX8, CBS* |
| **Complement** | Genes encoding components of the complement system, which is part of the innate immune system | *HSPA5, PLAUR, SERPINE1, CXCL1, DUSP5, COL4A2, CP, DUSP6, CSRP1, SH2B3, CR2, CALM1, USP14* |

**Sup. Table 6.** Representation of overlapping pathways/signaling of genes upregulated by siBRPF1 (The set of genes which lie in the intersection of the Venn diagram shown in **Sup. Figure 11A.**

| **Gene Set Name** | **Description** | **Genes** |
| --- | --- | --- |
| **Mitotic Spindle** | Genes important for mitotic spindle assembly | *FSCN1, TAOK2, ESPL1, RFC1, KIF15, BCL2L11, BIN1, DYNLL2, KLC1, TUBD1* |
| **Apical Junction** | Genes encoding components of apical junction complex | *FSCN1, TAOK2, PIK3R3, PTEN, MAP3K20, B4GALT1, GNAI2, CTNND1, LDLRAP1, PBX2* |
| **E2F Targets** | Genes encoding cell cycle related targets of E2F transcription factors | *ESPL1, RFC1, PPM1D, STMN1, CDC25B, MCM2, RAD21, NUP205, MXD3, TIMELESS* |
| **PI3K-AKT-mTOR Signaling** | Genes up-regulated by activation of the PI3K/AKT/mTOR pathway | *PIK3R3, PTEN, MYD88, AKT1, RAC1, UBE2N, DUSP3* |
| **IFNγ Response** | Genes up-regulated in response to IFNG [GeneID=3458]. | *MYD88, IFIT2, OGFR, IFI35, IFIT3, IFIT1, TNFAIP2, LATS2, SPPL2A* |
| **P53 Pathway** | Genes involved in p53 pathways and networks | *PPM1D, S100A10, RXRA, ACVR1B, S100A4, DDB2, MXD4, HEXIM1, PRMT2* |
| **G_2_M Checkpoint** | Genes involved in the G_2_/M checkpoint, as in progression through the cell division cycle | *ESPL1, KIF15, MAP3K20, STMN1, CDC25B, MCM2, RAD21, E2F2* |
| **Estrogen Response_Late** | Genes defining late response to estrogen. | *CCN5, BAG1, NBL1, CYP26B1, ACOX2, MEST, IMPA2* |
| **IFNα Response** | Genes up-regulated in response to alpha interferon proteins | *IFIT2, OGFR, IFI35, IFIT3, CNP* |
| **Androgen Response** | Genes defining response to androgens | *B4GALT1, AKT1, KRT8, H1-0, APPBP2* |
