# Supplementary material for: Exploiting epigenetic targets to overcome taxane resistance in prostate cancer
Source: Cell Death Dis. 2024 Feb 12;15(2):132. doi: 10.1038/s41419-024-06422-1 (PMC10861560; doi:10.1038/s41419-024-06422-1)
Supplement: Supplementary file 3 — Supplementary Figure and Table Legends [file 41419_2024_6422_MOESM3_ESM.docx]

**Supplementary Figure Legends**

**Sup. Figure 1.** **(A)** Timeline and schema for establishing taxane resistant cell models. Figure was made in BioRender. **(B)** The dose dependent effects of Dtx or Cbz on the cell viability of parental (P) and resistant (R) PCa cell lines. The results were obtained by using CTG assay (72 h) and expressed as mean ± SEM. (*) p < 0.01 and (**) p < 0.0001 indicate significant differences in cell viability between parental and resistant cells. **(C)** Representative clonogenic images were obtained by treating cells with Dtx or Cbz for 72 h and the colony formation ability was analyzed 10-15 days after drug exposure. **(D)** Flow cytometry analysis of cell death in taxane treated Du145/22Rv1-P and -R cells. **(E)** CETSA for in-cell β-Tubulin target engagement. Western blots showing thermostable β-Tubulin following indicated heat shocks in the presence of Dtx (62.5 nM) or Cbz (31.5 nM) in Du145-P/R cells. CETSA images were quantified by using ImageJ software (shown below). **P:** parental, **R:** resistant. Uncropped western blot images corresponding to **Sup. Figure 1E** were shown in **Supplemental Material.**

**Sup. Figure 2.** The dose dependent effects of **(A)** paclitaxel (Ptx), doxorubicin (Dox), **(B)** cisplatin (CisPt), carboplatin (CarboPt) and oxaliplatin (OxaPt) on the cell viability of parental and resistant PCa cells. The results were obtained by using SRB assay (72 h) and expressed as the mean ± SEM of triplicate from 2 biological replicates. The IC_50_ value of each cell line is shown in corresponding colors below the dose-response curves. (*) and (#) indicate significant differences in cell viability between parental and DtxR or CbzR cells, respectively (p < 0.05). **P:** parental, **R:** resistant.

**Sup. Figure 3. (A)** Gene set enrichment (GSEA) analysis using hallmark gene sets from the MSigDB in Du145-DCbzR cells (FDR < 0.05, and Log_2_FC ≥ 0.5 or ≤ -0.5). **(B)** Enrichment plots for the gene sets enriched in GSEA Hallmark analysis.

**Sup. Figure 4.** ABCB1 amplification in taxane resistant PCa cells. The data expressed as mean ± SEM from 2 biological replicates. (*) indicates significant differences ABCB1 gene copy numbers between parental and resistant cells (p < 0.0001).

**Sup. Figure 5.** The efficiency of siRNA mediated ABCB1 knockdown was evaluated by performing both **(A)** western blot and **(B)** qRt-PCR analyses. **(C)** The efficiency of ABCB1 knockout (CRISPR-Cas9) in 22Rv1-R cells was validated using Western blot and **(D)** qRt-PCR analysis. **(E, F)** Taxane response in ABCB1 silenced cells was assessed by SRB (72h) and clonogenic viability assays. **(G, H)** SRB (72h) and clonogenic viability assays were performed on gABCB1 and gNT treated 22Rv1-R cells. The data expressed as mean ± SEM. Statistical significance denoted as (*) p < 0.001 and (**) p < 0.0001. **(I)** Dose dependent effects of Epothilone B (Epo B), Dtx or Cbz on the cell viability of parental and taxane resistant Du145 and **(J)** 22Rv1 cells. The results were obtained by CTG assay (72h) and expressed as mean ± SEM. **P:** parental, **R:** resistant.

**Sup. Figure 6.** Validation dose-response of epidrugs (CBP30, MI2, HLCL61 and CAY10602) on taxane resistant PCa cells. **(A)** Cells were co-treated with indicated drugs (taxane; 1-250 nM and indicated inhibitors; 1.25-5 µM) and the results were obtained by SRB viability assay (72 h). The data is expressed as mean ± SEM. **(B)** Clonogenic images were obtained by treating cells with indicated drugs for 72 h and the colony formation ability was analyzed 10-15 days after drug exposure.

**Sup. Figure 7. (A)** Validation of SIRT overexpression by qRT-PCR, comparing non-transduced (NT) cells to stably SIRT1 expressing counterparts. The data is expressed as mean ± SEM. Statistical significance denoted as (*) p < 0.01 and (**) p < 0.001. **(B)** Taxane response of SIRT1-expressing cells was assessed by SRB viability assay (72h). **(C)** Knockdown efficiency of gCBP in Du145-DtxR cells. After transduction with control (gNT) and CBP-targeting gRNAs, the expression of CBP was detected by qRt-PCR. The colony formation ability of CBP-guided cells is represented under the corresponding column.

**Sup. Figure 8. (A)** Validation dose-response curves of BRPF inhibitors (PFI4, GSK5959 and OF1) on Cbz-resistant cells. Cells were co-treated with indicated drugs (Dtx; 1-250 nM and BRPF inhibitors; 1.25-5 µM) and the results were obtained by SRB viability assay (72 h). The data is expressed as mean ± SEM. **(C)** Cell cycle distribution (24h) in resensitized resistant cells. **(D)** Calcein retention assay was performed in the absence or presence of BRPF inhibitors (5 µM, 24h). **(E)** CETSA for in-cell ABCB1 engagement. Western blots showing thermostable ABCB1 following indicated heat shocks (44°C, 46°C, 48°C, 50°C and 52°C) in the presence of the indicated BRPF inhibitors (5 µM) in Du145-DtxR cells. **(F)** The efficacy of BRPF inhibitors on RPE-1 cells was evaluated using colony formation assay.

**Sup. Figure 9.** Viability graphs of non-targeting (NT) and ABCB1-targeting (gABCB1) guide received **(A)** Du145-DtxR and **(B)** 22Rv1-DtxR cells co-treated with BRPF inhibitors (5 µM) and Dtx. The results were obtained by CTG viability assay (72 h). The data is expressed as mean ± SEM.

**Sup. Figure 10. (A)** Colony forming capability of siRNA treated Du145-P/R cells. Representative images (left panel) and quantifications (right panel) are shown. **(B)** Efficiency and specificity of stable knockdown of BRPF1 in Du145-P and -DtxR cells. After transduction with control (shGPF) and shBRPF1, the expressions of BRPF1 and BRPF2 were detected by qRt-PCR. (*) p < 0.05 indicates significant differences between shGFP and shBRPF1 cells. **(C)** Five days after transduction, cells were analyzed for cell death (caspase 3/7 activity). Representative plots (left panel) and quantifications are shown (right panel).

**Sup. Figure 11. (A)** Venn diagram showing the number of genes (intersection, 291) whose expression increased after silencing of BRPF1 among genes with decreased expression in Du145-DtxR cells (vs Du145-P). **(B)** Computed overlaps of the 291 genes in the Hallmark Collection of GSEA (MSigDB) database.

**Sup. Figure 12.** Clonogenic images were obtained by treating Du145-P and -DtxR cells with Torin1 (mTORC1/2 inhibitor, 8-500 nM) and Ceapin-A7 (ATF6α inhibitor, 2.5-10 µM) for 72 h and the colony formation ability was analyzed 10-15 days after drug exposure.

**Sup. Figure 13. (A)** The Dtx response of Du145-DtxR cells was assessed after combining ABCB1 knockdown with Torin1 treatment (an mTORC1/2 inhibitor, 31-500 nM). The CTG viability assay was utilized, and the results were represented as a heat map. **(B)** Heat map representation of the Combination Index (CI) values, with red color indicating a synergistic effect. CI was calculated using the CalcuSyn software.

**Sup. Figure 14. (A)** The expression profiles of BRPF genes were analyzed by qRt-PCR, comparing taxane resistant cells to their parental counterparts. The data expressed as mean ± SEM from two biological replicates, each performed in duplicate. **(B)** The expression of BRPF genes in Du145-P cells was determined by qRt-PCR following Dtx treatment (1.25-5 nM) at the indicated time points. The data expressed as mean ± SEM. Statistical significance denoted as (*) p < 0.05, (**) p < 0.01, and (***) p < 0.001.

**Supplementary Table Legends**

**Sup. Table 1.** The nucleotide sequences of the primers used in the study.

**Sup. Table 2.** Top 10 ranked up- (red) and down-regulated (purple) genes in Du145-DtxR cells.

**Sup. Table 3.** Top 10 ranked up- (red) and down-regulated (purple) genes in Du145-CbzR cells.

**Sup. Table 4.** The genes in positively (NES ≥ 1.5) and negatively (NES ≤ 1.5) enriched gene sets in Du145-DtxR cells.

**Sup. Table 5.** Representation of overlapping pathways/signaling of genes downregulated by siBRPF1 (The set of genes which lie in the intersection of the Venn diagram shown in **Figure 4F.**

**Sup. Table 6.** Representation of overlapping pathways/signaling of genes upregulated by siBRPF1 (The set of genes which lie in the intersection of the Venn diagram shown in **Sup. Figure 11A.**
