# Supplementary material for: Exploiting epigenetic targets to overcome taxane resistance in prostate cancer
Source: Cell Death Dis. 2024 Feb 12;15(2):132. doi: 10.1038/s41419-024-06422-1 (PMC10861560; doi:10.1038/s41419-024-06422-1)

Image ID: 0000275_01
Acquire Time: 27.Eki.2023 10:48:21

Page 1

Acquisition Information

#	Image ID	Acquire Time	Channels	Integration Times	Analysis	Image Name	Comment	Image Modifications
1	0000275_01	27.Eki.2023 10:48:21	Cheml 700	00:30 00:30	Manual	0000275_01		

Image Display Values

Channel	Color	Minimum	Maximum	K
Cheml	Gray Scale (Black on White)	0,000207	0,0740	0
700	Gray Scale (Black on White)	0,0000407	0,0684	0

Figure 1-D-β-Tubulin-Du145

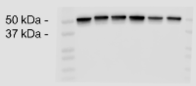

Image ID: 0000276_01
Acquire Time: 27.Eki.2023 10:52:57

Page 1

Acquisition Information

#	Image ID	Acquire Time	Channels	Integration Times	Analysis	Image Name	Comment	Image Modifications
1	0000276_01	27.Eki.2023 10:52:57	Cheml 700	00:30 00:30	Manual	0000276_01		

Image Display Values

Channel	Color	Minimum	Maximum	K
Cheml	Gray Scale (Black on White)	0,0000560	0,00690	0
700	Gray Scale (Black on White)	0,0000401	0,0605	0

Figure 1-D-ABCB1-Du145

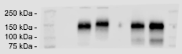

Image ID: 0000274_01
Acquire Time: 27.Eki.2023 10:43:47

Page 1

Acquisition Information

#	Image ID	Acquire Time	Channels	Integration Times	Analysis	Image Name	Comment	Image Modifications
1	0000274_01	27.Eki.2023 10:43:47	Cheml 700	00:30 00:30	Manual	0000274_01		

Image Display Values

Channel	Color	Minimum	Maximum	K
Cheml	Gray Scale (Black on White)	0,000235	0,0789	0
700	Gray Scale (Black on White)	0,0000433	0,0990	0

Figure 1-D-β-Tubulin-22Rv1

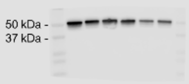

Image ID: 0000273_01
Acquire Time: 27.Eki.2023 10:37:55

Page 1

Acquisition Information

#	Image ID	Acquire Time	Channels	Integration Times	Analysis	Image Name	Comment	Image Modifications
1	0000273_01	27.Eki.2023 10:37:55	Cheml 700	00:30 00:30	Manual	0000273_01		

Image Display Values

Channel	Color	Minimum	Maximum	K
Cheml	Gray Scale (Black on White)	0,00000346	0,00975	0
700	Gray Scale (Black on White)	0,000119	0,102	0

Figure 1-D-ABCB1-22Rv1

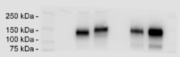

LI-COR

Image ID: 0009889_01
Acquire Time: 22.Nis.2022 13:22:15

Acquisition Information

#	Image ID	Acquire Time	Channels	Integration Times	Analysis	Image Name	Comment	Image Modifications
1	0009889_01	22.Nis.2022 13:22:15	Chemi	00:30	Western	0009889_01		

Image Display Values

Channel	Color	Minimum	Maximum	K
Chemi	Gray Scale (Black on White)	0,00000370	0,0113	0

Figure 2-F-GSK5959-H3total

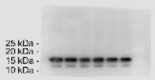

LI-COR

Image ID: 0009810_01
Acquire Time: 19.Nis.2022 14:39:03

Acquisition Information

#	Image ID	Acquire Time	Channels	Integration Times	Analysis	Image Name	Comment	Image Modifications
1	0009810_01	19.Nis.2022 14:39:03	Chemi	00:30	Western	0009810_01		

Image Display Values

Channel	Color	Minimum	Maximum	K
Chemi	Gray Scale (Black on White)	0,00000358	0,0295	0

Figure 2-F-GSK5959-H3K27Ac

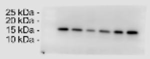

LI-COR

Image ID: 0009808_01
Acquire Time: 19.Nis.2022 14:34:03

Acquisition Information

#	Image ID	Acquire Time	Channels	Integration Times	Analysis	Image Name	Comment	Image Modifications
1	0009808_01	19.Nis.2022 14:34:03	Chemi	00:30	Western	0009808_01		

Image Display Values

Channel	Color	Minimum	Maximum	K
Chemi	Gray Scale (Black on White)	0,00000864	0,225	0

Figure 2-F-GSK5959-H3K18Ac

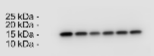

LI-COR

Image ID: 0009806_01
Acquire Time: 19.Nis.2022 14:25:35

Acquisition Information

#	Image ID	Acquire Time	Channels	Integration Times	Analysis	Image Name	Comment	Image Modifications
1	0009806_01	19.Nis.2022 14:25:35	Chemi	00:30	Western	0009806_01		

Image Display Values

Channel	Color	Minimum	Maximum	K
Chemi	Gray Scale (Black on White)	0,00000435	0,0724	0

Figure 2-F-GSK5959-H3K9Ac

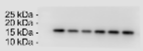

LI-COR

Image ID: 0009442_01
Acquire Time: 01.Mar.2022 13:52:26

Acquisition Information

#	Image ID	Acquire Time	Channels	Integration Times	Analysis	Image Name	Comment	Image Modifications
1	0009442_01	01.Mar.2022 13:52:26	Cheml	00:30	Western	0009442_01		

Image Display Values

Channel	Color	Minimum	Maximum	K
Cheml	Gray Scale (Black on White)	0,00000352	0,0161	0

Figure 2-F-OF1-H3total

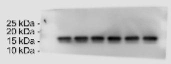

LI-COR

Image ID: 0009452_01
Acquire Time: 01.Mar.2022 14:27:48

Acquisition Information

#	Image ID	Acquire Time	Channels	Integration Times	Analysis	Image Name	Comment	Image Modifications
1	0009452_01	01.Mar.2022 14:27:48	Cheml	00:30	Western	0009452_01		

Image Display Values

Channel	Color	Minimum	Maximum	K
Cheml	Gray Scale (Black on White)	0,00000334	0,0132	0

Figure 2-F-OF1-H3K27Ac

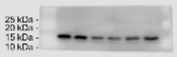

LI-COR

Image ID: 0009453_01
Acquire Time: 01.Mar.2022 14:32:55

Acquisition Information

#	Image ID	Acquire Time	Channels	Integration Times	Analysis	Image Name	Comment	Image Modifications
1	0009453_01	01.Mar.2022 14:32:55	Cheml	00:30	Western	0009453_01		

Image Display Values

Channel	Color	Minimum	Maximum	K
Cheml	Gray Scale (Black on White)	0,00000381	0,0179	0

Figure 2-F-OF1-H3K18Ac

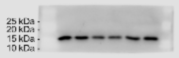

LI-COR

Image ID: 0009446_01
Acquire Time: 01.Mar.2022 14:03:35

Acquisition Information

#	Image ID	Acquire Time	Channels	Integration Times	Analysis	Image Name	Comment	Image Modifications
1	0009446_01	01.Mar.2022 14:03:35	Cheml	00:30	Western	0009446_01		

Image Display Values

Channel	Color	Minimum	Maximum	K
Cheml	Gray Scale (Black on White)	0,000156	0,0598	0

Figure 2-F-OF1-H3K9Ac

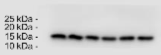

Image ID: 0009724_01
Acquire Time: 06.Nis.2022 14:26:03

Page 1

Acquisition Information

#	Image ID	Acquire Time	Channels	Integration Times	Analysis	Image Name	Comment	Image Modifications
1	0009724_01	06.Nis.2022 14:26:03	Chemi	00:30	Western	0009724_01		

Image Display Values

Channel	Color	Minimum	Maximum	K
Chemi	Gray Scale (Black on White)	0,00000298	0,0134	0

Figure 2-F-PFI4-H3total

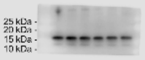

Image ID: 0009723_01
Acquire Time: 06.Nis.2022 14:23:10

Page 1

Acquisition Information

#	Image ID	Acquire Time	Channels	Integration Times	Analysis	Image Name	Comment	Image Modifications
1	0009723_01	06.Nis.2022 14:23:10	Chemi	00:30	Western	0009723_01		

Image Display Values

Channel	Color	Minimum	Maximum	K
Chemi	Gray Scale (Black on White)	0,00000268	0,0131	0

Figure 2-F-PFI4-H3K27Ac

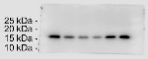

Image ID: 0009812_01
Acquire Time: 19.Nis.2022 14:45:28

Page 1

Acquisition Information

#	Image ID	Acquire Time	Channels	Integration Times	Analysis	Image Name	Comment	Image Modifications
1	0009812_01	19.Nis.2022 14:45:28	Chemi	00:30	Western	0009812_01		

Image Display Values

Channel	Color	Minimum	Maximum	K
Chemi	Gray Scale (Black on White)	0,0000832	0,0593	0

Figure 2-F-PFI4-H3K18Ac

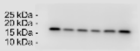

Image ID: 0009721_01
Acquire Time: 06.Nis.2022 14:17:43

Page 1

Acquisition Information

#	Image ID	Acquire Time	Channels	Integration Times	Analysis	Image Name	Comment	Image Modifications
1	0009721_01	06.Nis.2022 14:17:43	Chemi	00:30	Western	0009721_01		

Image Display Values

Channel	Color	Minimum	Maximum	K
Chemi	Gray Scale (Black on White)	0,00000370	0,0512	0

Figure 2-F-PFI4-H3K9Ac

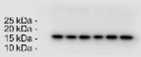

Image ID: 0011723_01
Acquire Time: 01.Ara.2022 11:19:47

Acquisition Information

#	Image ID	Acquire Time	Channels	Integration Times	Analysis	Image Name	Comment	Image Modifications
1	0011723_01	01.Ara.2022 11:19:47	Chemi	00:30	Manual	0011723_01		

Image Display Values

Channel	Color	Minimum	Maximum	K
Chemi	Gray Scale (Black on White)	0,00000972	0,0171	0

Figure 3-H-Zosu-ABCB1

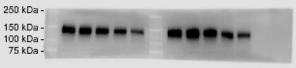

Image ID: 0011722_01
Acquire Time: 01.Ara.2022 11:16:28

Acquisition Information

#	Image ID	Acquire Time	Channels	Integration Times	Analysis	Image Name	Comment	Image Modifications
1	0011722_01	01.Ara.2022 11:16:28	Chemi	00:30	Manual	0011722_01		

Image Display Values

Channel	Color	Minimum	Maximum	K
Chemi	Gray Scale (Black on White)	0,00000477	0,0182	0

Figure 3-H-GSK5959-ABCB1

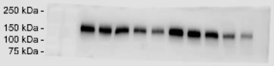

Image ID: 0012302_01
Acquire Time: 20.Oca.2023 14:07:42

Page 1

Acquisition Information

#	Image ID	Acquire Time	Channels	Integration Times	Analysis	Image Name	Comment	Image Modifications
1	0012302_01	20.Oca.2023 14:07:42	Chemi	00:30	Manual	0012302_01		

Image Display Values

Channel	Color	Minimum	Maximum	K
Chemi	Gray Scale (Black on White)	0,00000322	0,00592	0

Sup. Figure 5-A-GAPDH

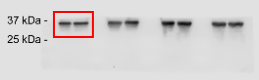

Image ID: 0012303_01
Acquire Time: 20.Oca.2023 14:10:38

Page 1

Acquisition Information

#	Image ID	Acquire Time	Channels	Integration Times	Analysis	Image Name	Comment	Image Modifications
1	0012303_01	20.Oca.2023 14:10:38	Chemi 700	00:30 00:30	Manual	0012303_01		

Image Display Values

Channel	Color	Minimum	Maximum	K
Chemi	Gray Scale (Black on White)	0,00000328	0,0225	0
700	Gray Scale (Black on White)	0,000131	0,0488	0

Sup. Figure 5-A-ABCB1

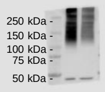

Image ID: 0000887_01
Acquire Time: 23.May.2023 15:09:23

Acquisition Information

#	Image ID	Acquire Time	Channels	Integration Times	Analysis	Image Name	Comment	Image Modifications
1	0000887_01	23.May.2023 15:09:23	Chemi	00:30	Manual	0000887_01		

Image Display Values

Channel	Color	Minimum	Maximum	K
Chemi	Gray Scale (Black on White)	0,00000435	0,0329	0

Sup. Figure 5-C-GAPDH

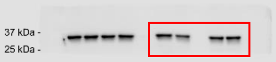

Image ID: 0000890_01
Acquire Time: 23.May.2023 15:24:59

Acquisition Information

#	Image ID	Acquire Time	Channels	Integration Times	Analysis	Image Name	Comment	Image Modifications
1	0000890_01	23.May.2023 15:24:59	Chemi 700	00:30 00:30	Manual	0000890_01		

Image Display Values

Channel	Color	Minimum	Maximum	K
Chemi	Gray Scale (Black on White)	0,000112	0,0321	0
700	Gray Scale (Black on White)	0,000151	0,0292	0

Sup. Figure 5-C-ABCB1

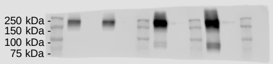

Image ID: 0011762_01
Acquire Time: 05.Ara.2022 10:34:48

Acquisition Information

#	Image ID	Acquire Time	Channels	Integration Times	Analysis	Image Name	Comment	Image Modifications
1	0011762_01	05.Ara.2022 10:34:48	Chemi	00:30	Manual	0011762_01		

Image Display Values

Channel	Color	Minimum	Maximum	K
Chemi	Gray Scale (Black on White)	0,00000429	0,00986	0

Sup. Figure 8-E-OF1-ABCB1

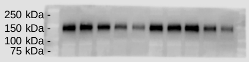

Image ID: 0011763_01
Acquire Time: 05.Ara.2022 10:38:04

Acquisition Information

#	Image ID	Acquire Time	Channels	Integration Times	Analysis	Image Name	Comment	Image Modifications
1	0011763_01	05.Ara.2022 10:38:04	Chemi	00:30	Manual	0011763_01		

Image Display Values

Channel	Color	Minimum	Maximum	K
Chemi	Gray Scale (Black on White)	0,00000525	0,00657	0

Sup. Figure 8-E-PFI4-ABCB1

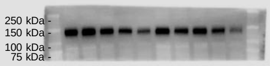

Supplement: Supplementary file 4 — Supplementary Data-Uncropped WB Images [file 41419_2024_6422_MOESM4_ESM.pdf]
